# Functional footprints of homologous recombination deficiency in prostate cancer revealed by ctDNA fragmentation and transcription factor accessibility

**DOI:** 10.1038/s41416-025-03301-0

**Published:** 2026-01-09

**Authors:** Georgios Vlachos, Tina Moser, Isaac Lazzeri, Matthias J. Moser, Lisa Glawitch, Emil Thomas Bauernhofer, Anna Eberhard, Christine Beichler, Hanieh Sadeghi, Jasmin Blatterer, Stefan Kühberger, Nina Monsberger, Angelika Terbuch, Karl Kashofer, Jochen B. Geigl, Thomas Bauernhofer, Ellen Heitzer

**Affiliations:** 1 Institute of Human Genetics, Diagnostic & Research Center for Molecular BioMedicine, Medical University of Graz, Graz, Austria; 2 Christian Doppler Laboratory for Liquid Biopsies for Early Detection of Cancer, Medical University of Graz, Medical University of Graz, Graz, Austria; 3 Department of Internal Medicine Graz, Division of Oncology, Medical University of Graz, Graz, Austria; 4 Diagnostic and Research Institute of Pathology, Medical University of Graz, Graz, Austria

**Keywords:** Diagnostic markers, Cancer epigenetics

## Abstract

**Background:**

Homologous recombination deficiency (HRD) is a predictive biomarker for response to PARP inhibitors and platinum-based therapies in prostate cancer (PCa). However, current diagnostic approaches, often limited to *BRCA1/2* mutation testing or genomic scars, fail to capture the full spectrum of HRD. Tissue-based testing is further hampered by tumour heterogeneity and biopsy limitations in patients with metastatic bone disease. This study aimed to develop a noninvasive, multimodal ctDNA-based strategy for comprehensive HRD profiling in advanced PCa.

**Methods:**

We analysed plasma-derived ctDNA from 106 patients with metastatic PCa. The approach integrated targeted sequencing of homologous recombination repair (HRR) genes, low-pass whole genome sequencing for genomic instability scores (GIS), whole-exome sequencing for mutational signature analysis, and cfDNA fragmentomics, including chromatin accessibility profiling.

**Results:**

*BRCA2* was the most frequently altered HRR gene, frequently co-occurring with *PTEN* loss. High GIS was associated with *BRCA2*/*RB1* loss, increased somatic copy number alterations, and poor overall survival. HRD tumours were enriched for mutational signatures SBS3 and ID6, displayed increased dinucleosome-length fragments, and showed reduced accessibility at zinc finger transcription factor binding sites. A fragmentomics-based classifier identified HRD-positive cases with high accuracy.

**Conclusions:**

Our findings support the use of multimodal ctDNA profiling as a non-invasive approach to identify HRD in prostate cancer. The integration of mutation, genomic instability, and fragmentomic features provides a broader functional view of HRD and may enhance patient stratification for targeted therapies.

## Background

Prostate cancer (PCa) is the second most common cancer in men, and metastatic castration-resistant PCa (mCRPC) remains a leading cause of cancer-related death [1]. A key vulnerability in PCa is homologous recombination deficiency (HRD), present in 5–10% of localised cases and up to 25% of metastatic cases, depending on detection methods [2–6]. HRD results from germline or somatic mutations in homologous recombination repair (HRR) genes (e.g.*, BRCA2*, *BRCA1*, and *PALB2*), epigenetic silencing, and dysregulation of transcriptional and post-translational pathways [3, 7]. HRD leads to impaired DNA repair, genomic instability (GIS), somatic copy number alterations (SCNAs), and characteristic mutational signatures (e.g., SBS3). However, specifically in PCa, integrated whole-genome sequencing (WGS) analyses have revealed that 5–8% of localised cases exhibit HRD-associated mutational signatures independent of *BRCA1/2* mutations, expanding potential PARP inhibitor eligibility [4, 5]. Therefore, relying solely on mutation screening may overlook the cases caused by other mechanisms.

Clinically, HRD in PCa has been linked to more aggressive disease phenotypes, earlier onset, but increased sensitivity to DNA-damaging agents, such as PARP inhibitors (PARPi) and platinum-based chemotherapies. Resistance can emerge through reversion mutations or restoration of HRR function [8], underscoring the need for an accurate and dynamic assessment of HRD status. However, in bone-predominant metastatic PCa, tissue biopsies are often challenging, making circulating tumour DNA (ctDNA) a valuable, non-invasive alternative that captures both spatial and temporal tumour heterogeneity [9, 10]. Nonetheless, ctDNA analysis is limited by the absence of matched germline DNA, which complicates distinguishing somatic from inherited variants and increases the risk of false positives related to clonal hematopoiesis (CH) [11].

Recent studies have shown that cfDNA fragmentation features—including size profiles, end motifs, and nucleosome positioning—carry significant diagnostic value across cancers [12–14]. Given their elevated GIS, HRD phenotypes likely extend beyond canonical mutations, and HRD tumours may also display distinct fragmentation and chromatin accessibility patterns in cfDNA, informing both molecular subtyping and HRD detection. Therefore, single-modality assays may underestimate HRD prevalence, while multimodal approaches integrating mutations, SCNAs, genomic scars, and fragmentomics features may offer superior detection of intrinsic tumour biology [15–17].

**Fig. 1:**
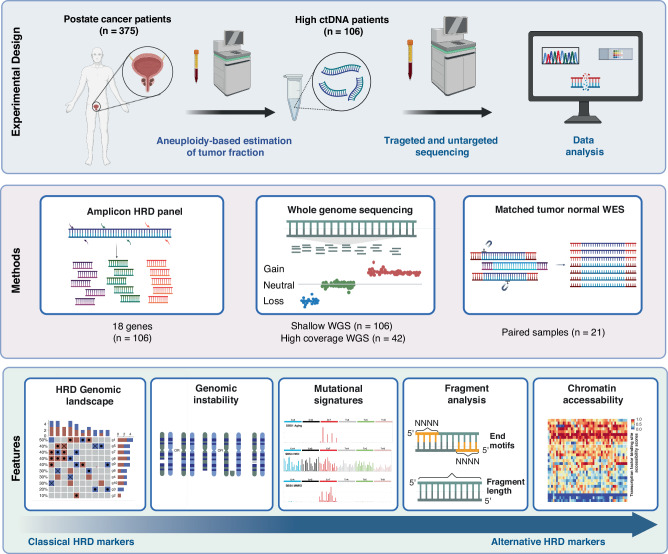
Multimodal framework for HRD detection from cfDNA in advanced prostate cancer. From an initial cohort of 375 patients with prostate cancer with multiple sampling time points, we selected samples with elevated ctDNA levels from 106 patients for in-depth analysis. A tiered approach was applied to characterise both the causes and consequences of homologous recombination deficiency (HRD). Targeted panel sequencing was used to assess pathogenic variants in 18 HRR-related genes. Low-pass and high-coverage whole-genome sequencing (WGS) enabled the quantification of genomic instability and the extraction of fragmentomics and chromatin accessibility features. Whole-exome sequencing (WES) of matched tumour-normal cfDNA samples was performed in a subset to characterise HRD-associated mutational signatures. Downstream analyses integrated genomic alterations, genomic instability scores, mutational signatures, fragment length patterns, end motifs, and transcription factor binding site (TFBS) accessibility to derive classical and alternative HRD biomarkers from plasma. Created in BioRender. Heitzer, E. (2025) https://BioRender.com/zhwwhzq.

We employed a tiered multimodal strategy to characterise the causes and consequences of HRD in metastatic prostate cancer using cfDNA (Fig. 1). Subsequent layers of investigation included: (i) targeted amplicon sequencing of HRR pathway genes, (ii) whole exome sequencing (WES) for mutational signature analysis, and (iii) WGS to quantify large-scale genomic scars. In addition, we explored fragmentomics features and chromatin accessibility at transcription factor binding sites (TFBS). This approach enabled the robust characterisation of HRD-associated features in a clinically relevant cohort and demonstrated the potential of cfDNA as a scalable, non-invasive analyte for functional HRD assessment in prostate cancer beyond genetic alterations.

## Methods

Detailed methods are presented in the Supplementary Materials.

### Sample collection and cfDNA isolation

Between 2012 and 2023, 898 plasma samples were collected from 375 patients with metastatic prostate cancer at the Medical University of Graz, Austria. Blood samples were collected in Cell-Free DNA blood collection tubes (Streck) or PAXgene Blood ccfDNA Tubes (PreAnalytiX) at the Division of Oncology at the Medical University of Graz, Austria. Plasma was separated via double-spin centrifugation; cfDNA was extracted from 2 mL plasma using the QIAamp Circulating Nucleic Acid Kit (QIAGEN) and quantified with the Qubit 4 Fluorometer (ThermoFisher) [18].

### Modified Fast Aneuploidy Screening Test-Sequencing System (mFAST-SeqS)

Aneuploidy and tumour fraction were estimated using the modified FAST-SeqS method [19]. Briefly, LINE-1 elements were PCR-amplified from 1 ng of cfDNA, followed by indexing, library purification, and sequencing (Illumina, 100k reads). Reads were aligned to hg19 using BWA-MEM2 (RRID: SCR_022192, v.0.7.4) [20], and Z-scores were calculated per chromosome arm against 35 healthy controls to assess genome-wide copy number alterations.

### Panel analysis of clinically relevant genes

A QIAseq HRR panel was used to detect mutations in key DNA repair genes included in the PROfound study [21], and expanded to RB1, PTEN, and TP53, given their well-established clinical relevance in prostate cancer progression and neuroendocrine differentiation (see Supplementary Material for full gene list). Assay sensitivity was validated with Seraseq ctDNA standards at varying VAFs (0.1–5%) (Supplementary Data) and revealed a limit of detection (LOD) of 0.5%. Libraries were prepared from 10–40 ng cfDNA using UMIs and sequenced on Illumina platforms (≥25,600× coverage). Reads were aligned to hg19; UMIs were clustered with smCounter2 [22–24]. Variants were annotated in VarSeq using gnomAD and dbNSFP and classified per ACMG-AMP guidelines [25].

### Co-occurrence analysis

The calculation of expected co-occurrence is based on the assumption that two alterations occur independently. For each alteration A, the probability P(A) was calculated by dividing the number of patients with the alteration by the total number of patients. The expected number of co-occurrences between alterations A and B is the product of their individual probabilities multiplied by the Total Number of Patients expected to co-occur (A, B) = P(A) × P(B) × Total Number of Patients [26].

### WES library preparation and sequencing

WES was performed on 21 cfDNA tumour samples and matched germline DNA. cfDNA libraries used Illumina DNA Prep with Twist Exome 2.5; germline libraries used Twist Human Exome 2.0. Sequencing was done on NovaSeq 6000 (2 × 150 bp). Tumour samples had >1500× average coverage; germline samples had mean 150×. Using Twist cfDNA reference standards, sensitivity was >94% for 1% VAF variants (Supplementary Fig. 8)*.*

### Mutational signature analysis

Mutational signatures were called using the MutationalPatterns (RRID: SCR_024247) package with the strict refit method, utilising COSMIC (RRID: SCR_002260, v3.4) for signature refitting [27–29]. Data normality was tested with the Shapiro-Wilk; group differences were evaluated using two-sided ANOVA and two-sided Mann-Whitney U tests (*p* < 0.05).

### Whole-genome sequencing

Low-pass WGS (lpWGS, ~0.1–0.2×) was done on 106 cfDNA samples and high-coverage WGS (hcWGS, ~30×) on 42. Libraries (10 ng input) were prepared with TruSeq DNA Nano Kit [30], pooled, and sequenced on NovaSeq 6000. BAMs were aligned to hg19; duplicates marked using Picard [31]. CNAs were called from lpWGS using ichorCNA [32], and events <5 bins or below tumour fraction thresholds were excluded. Genomic loci were mapped using UCSC genome data [33]. Genomic instability was assessed using the shallowHRD tool (sHRD) [34]. Samples were categorised as HRD+ or HRD-based on large-scale genomic alterations. Structural variant (SV) analysis of hcWGS data was performed using MANTA [35]. To correct for potential GC bias in our fragmentomics analyses, we applied GCparagon, a method specifically developed for fragment length-specific GC correction in WGS cfDNA datasets [36].

### Fragment feature analysis

Filtered BAMs were used to extract cfDNA fragment lengths (80–400 bp) and 5′ end 4-mer motifs across the genome without restriction to regions with copy number changes. Length profiles were smoothed with 32 bp rolling averages and standardised as Z-scores. End-motif frequencies were CLR-transformed and clustered (Spectral Clustering); rare clusters were excluded, yielding 19 key features. Given the limited number of HRD-positive cases, we applied 100-fold stratified cross-validation using L2-penalised logistic regression (C = 1) with late fusion of probability scores. Training was restricted to samples with pathogenic *BRCA1/2* or *PALB2* mutations, while cases with high sHRD scores or HRD-associated signatures were excluded to reduce label noise. To ensure robustness in this small cohort, we used a highly specific HRD-positive definition and derived all features from control data to avoid leakage, enabling stable and interpretable cfDNA-based HRD classification.

### Transcription factor binding analysis

LBFextract was used to quantify chromatin accessibility at TFBS in cfDNA using high-confidence TFBS coordinates from the Gene Transcription Regulation Database (GTRD v21.12, hg38) [37]. Normalised coverage was calculated across ±2000 bp windows around TFBS. Group differences were tested using the two-sided Mann-Whitney U test with Benjamini-Hochberg correction; outliers were removed, and TFs with FDR < 0.05 were retained. Samples were stratified into *BRCA2*/sHRD-high, *TP53*/*RB1*-altered or neuroendocrine (NE), and HRR-proficient groups, with comutated cases assigned to the *TP53*/*RB1*/NE category due to their stronger lineage plasticity and NE signalling. For details, see Supplementary material.

## Results

### Selection of PCa samples suitable for cfDNA-based inference of HRD

The primary goal of our study was to characterise HRD at the genetic and functional levels. To achieve sufficient analytical depth for genomic and fragmentomics profiling, we enriched for samples with high ctDNA content, independent of their known HRD status at the time of selection using mFAST-SeqS and lpWGS **(**Supplementary Fig. 1a**)** [19, 32]. Among 898 plasma samples from 375 prostate cancer patients, 257 (28.6%) had a Z-score >5, indicating TFs of ~5–10%. From this group, 82 high-TF cases (median Z-score 34.3) and 24 additional lpWGS-identified cases were selected (total *n* = 106), all with sufficient plasma for targeted sequencing and ichorCNA-based lpWGS (median TF 30.26%, range 0–80.34%) **(**Supplementary Fig. 1b, c**)**.

Clinical data were available for 102 patients, including mCRPC (*n* = 60), mHSPC (*n* = 33), neuroendocrine PCa (*n* = 7), and unclassified (*n* = 2). As expected, neuroendocrine PCa showed the poorest survival **(**Supplementary Fig. 2**)**. The median sampling age was 65 years (range 47–88). Most patients had metastatic disease and maintained high Karnofsky scores despite advanced cancer (Supplementary Table 1**)**. TF correlated strongly with Z-scores and variant allele frequencies (VAF) **(**Supplementary Fig. 3**)**, with the highest TFs observed in mCRPC and neuroendocrine cases **(**Supplementary Fig. 1d, e).

### Patterns of pathogenicity, co-occurrence, and clonality of HRD alterations

To characterise the causes of HRD in ctDNA, we sequenced 18 genes involved in HRR, as well as key tumour suppressors associated with poor prognosis and therapy resistance*.* Pathogenic mutations were detected in 65 patients (61.3%) **(**Supplementary Fig. 4), including *BRCA1/2* or *PALB2* mutations in 14 (13.2%) and other HRR gene alterations in 13 patients (12.3%), respectively. *TP53* (43.4%), *RB1* (43.4%), *PTEN* (9.4%) mutations were more frequent, while *CDK12* mutations were identified in only 3.8% of patients.

**Fig. 2:**
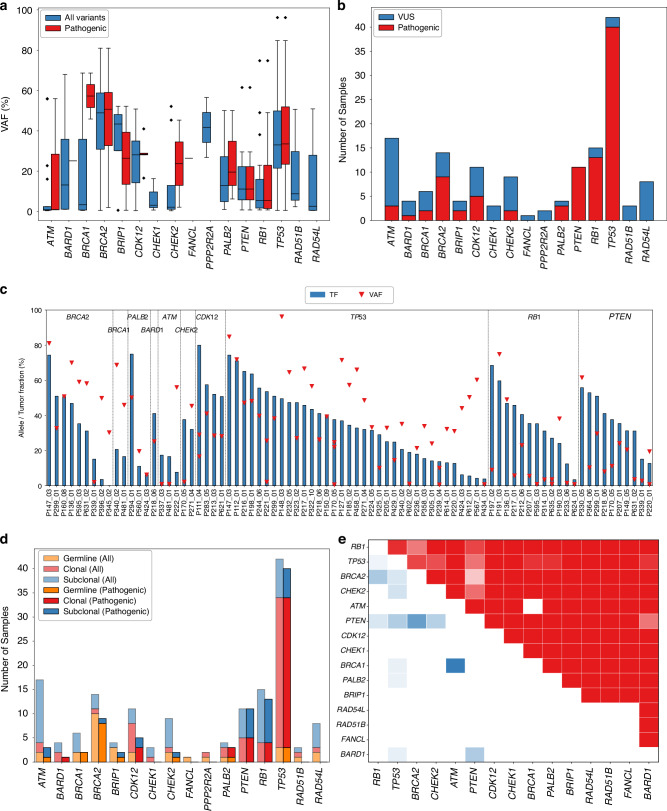
Patterns of pathogenicity, clonality, and co-occurrence of HRD-associated alterations in cfDNA. **a** Variant allele frequencies (VAFs) of mutations detected in HRR genes and key tumour suppressors (*TP53*, *RB1, PTEN*). **b** Distribution of variant classifications across genes. **c** Shown are tumour fractions (TF, blue bars) and VAF of detected mutations for clonality assessment of mutations. **d** Distribution of mutational origin. **e** Co-occurrence matrix of identified mutations; positive co-occurrence (blue): significantly co-occur; negative co-occurrence (red).

Non-HRR genes harboured mostly high-VAF clonal mutations, while HRR genes showed broader VAF distributions, reflecting greater subclonality or germline contribution **(**Fig. 2a). Pathogenicity rates were higher for non-HRR genes (94.1%) than for HRR genes (33.9%, *p* < 0.0001), with *BRCA2* and *ATM* showing a higher proportion of VUS **(**Fig. 2b). Most pathogenic variants were clonal, whereas subclonal mutations, mainly in *TP53*, *ATM*, and *CHEK2 -* were often below the expected VAFs, suggesting intratumoral heterogeneity or clonal hematopoiesis **(**Fig. 2c)*.*

Germline variants were enriched in HRR genes, particularly *BRCA2* (70.6% germline), with others in *ATM*, *BRCA1*, *BRIP1*, and *RAD54L*, whereas *TP53*, *RB1*, and *PTEN* mutations were predominantly somatic (Fig. 2d)*.* Co-occurrence analysis showed *BRCA2* frequently co-occurred with *PTEN* and *RB1*, whereas *TP53* showed a pattern of reduced co-occurrence with both, indicating distinct molecular subtypes. *CDK12* mutations were also mutually exclusive with other alterations, which is consistent with their unique genomic profile in PCa (Fig. 2e*,* Supplementary Table 3).

### Copy number profiling reveals complementary information about HRR status

**Fig. 3:**
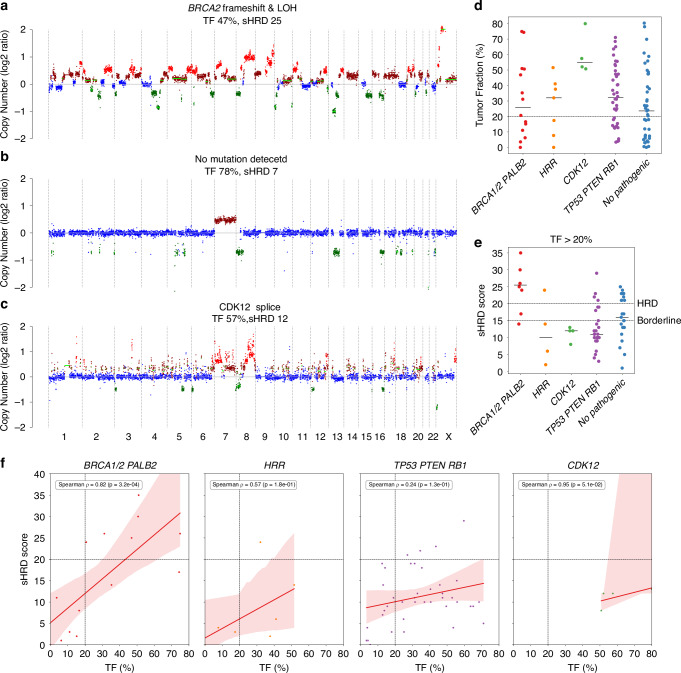
Genomic instability and copy number features associated with HRD in cfDNA. **a** Representative copy number profile of a *BRCA2*-mutated (HRD) sample. **b** Representative copy number profile of a HR-proficient prostate cancer samples. **c** Distinct focal tandem duplications (FTDs), characteristic of *CDK12*-mutated tumours. **d** Distribution of tumour fraction (TF%) across five mutation-defined groups: *BRCA1*/2 or *PALB2*, non *BRCA/PALB2* HRR genes, *CDK12, TP53/PTEN/RB1*, and samples without pathogenic mutations. **e** ShallowHRD scores (sHRD) in the same groups, limited to samples with TF > 20%. Dashed lines indicate sHRD thresholds of 15 and 20. **f** Correlation between TF% and sHRD score within each genomic subgroup. Dashed lines mark TF = 20% and sHRD = 20. Spearman correlation statistics are reported per panel.

Copy number profiling revealed marked differences between HRR-deficient and -proficient prostate cancers. *BRCA1/2*-mutated samples showed highly fragmented genomes and elevated GIS, while HRR-proficient tumours remained relatively stable across TF levels (Fig. 3a, b). Even monoallelic HRR gene losses were associated with increased SCNAs, underscoring clinical relevance beyond the two-hit model [38]. *CDK12*-mutated tumours displayed distinct focal tandem duplications (FTDs), a hallmark of *CDK12* inactivation, absent in VUS cases (Fig. 3c). Unlike *BRCA*-related HRD, *CDK12* loss may drive oncogenesis via gene fusion and neoantigen production, potentially favouring immunotherapy over PARPi [39].

GIS quantified using sHRD [34] strongly correlated with TF in HRR-mutated tumours, confirming HRD specificity. *BRCA1/2/PALB2*-mutated samples showed consistently high sHRD scores, especially in high-TF samples (Fig. 3d, e). Other subtypes showed lower or variable scores, indicating distinct GIS mechanisms. High-sHRD cases were enriched for deletions at loci such as 13q14.2 (*RB1*), 10q23.2 (*PTEN*), 11q23.2 (*ATM*), and amplifications at 8q24.13 (*MYC*/*NBN*) (Supplementary Fig. 5). Notably, 5q15 deletions not previously linked to HRD may represent novel instability loci.

*RB1* loss (including gross deletions) was the most frequent alteration, while *BRCA2* mono- or biallelic loss was most enriched in high-sHRD tumours (Supplementary Fig. 4). Other HRR mutations showed weaker sHRD correlations, reinforcing the central role of structural variation and aneuploidy in driving genomic instability [40]. sHRD scores were significantly associated with *BRCA2* (*p* = 0.0413) and *RB1* (*p* = 0.0765) alterations **(**Supplementary Fig. 6a), highlighting their key roles in GIS, whereas sHRD scores of *TP53* mutated samples were more broadly distributed.

Complementing these results, SV analysis of hcWGS data identified 7,171 coding-region events, predominantly large deletions. Although no recurrent HRR gene fusions were detected, multi-gene deletions, frequently involving *BRCA1/2*, *PALB2*, *RAD51B/C/D*, *FANCD2*, *FANCM*, *ATR*, and *CDK12*, indicate that large-scale genomic losses are a major structural mechanism of HRD in prostate cancer (Supplementary Fig. 13).

### Clinical implications of sHRD and key genomic alterations on survival

**Fig. 4:**
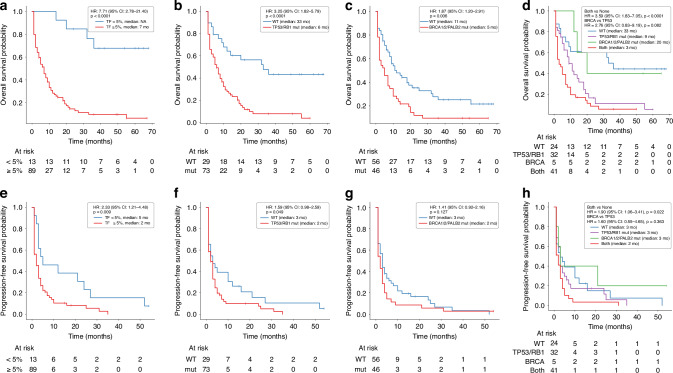
Survival analysis by ctDNA burden and HRD-related mutations in prostate cancer. Overall survival (OS) of patients stratified by **a** tumour fraction (TF), **b** presence of *TP53* or *RB1* mutations, **c** presence of *BRCA/PALB2* mutations, and **d** no pathogenic mutations detected, detected *TP53/RB1* mutations, detected *BRCA/PALB2* only, and detected *TP53/RB1/BRCA/PALB2* mutations. Progression-free survival (PFS) of patients stratified by **e** tumour fraction (TF), **f** presence of *TP53* or *RB1* mutations, **g** presence of *BRCA/PALB2* mutations, and **h** no pathogenic mutations detected, detected *TP53/RB1* mutations, detected *BRCA/PALB2* only, and detected *TP53/RB1/BRCA/PALB2* mutations. Median survival times are indicated in the legend. Hazard ratios (HR), 95% confidence intervals (CI), and log-rank *p*-values were derived from Cox proportional hazards models and pairwise log-rank tests. Censoring is shown with vertical tick marks, and at-risk counts are displayed below each curve.

To assess the clinical relevance of pathogenic alterations, GIS, and TF in patient outcomes, we performed Kaplan-Meier survival analyses **(**Fig. 4). Patients with TF ≥ 5% had significantly worse overall survival (OS) compared to those with lower TF (median 7 vs. not reached months, HR 7.71, *p* < 0.0001, Fig. 4a). Progression-free survival (PFS) was also shorter in patients with high TF (median 2 vs. 5 months, HR 2.33, *p* = 0.009; Fig. 4b), reflecting the impact of tumour burden on prognosis.

Stratification by sHRD score revealed that patients with sHRD < 20 had a significantly longer OS (median 10 vs. 3 months, HR 2.4, *p* = 0.001, Supplementary Fig. 7a), although sHRD did not correlate with PFS (HR 1.14, *p* = 0.67; Supplementary Fig. 7b). This suggests that sHRD reflects long-term survival potential rather than an immediate treatment response. *TP53* and/or *RB1* mutations or deletions were associated with poor OS (median, 6 vs. 33 months; HR, 3.25; *p* < 0.0001; Fig. 4b). Similarly, *BRCA1/2* or *PALB2* alterations were associated with shorter OS (median 5 vs. 11 months, HR 1.87, *p* = 0.006, Fig. 4c), although this did not significantly affect PFS, likely because of sample size or treatment variation. To further investigate the interaction between these pathways, we performed a combined mutation survival analysis, stratifying the patients into four groups: (i) no pathogenic mutations, (ii) *TP53/RB1* only, (iii) *BRCA1/2/PALB2* only, and iv) both mutation types (Fig. 4d). Those with no mutations had the best OS (median 33 months), followed by *BRCA*-only (20 months), *TP53/RB1*-only (9 months), and combined mutation cases, which had the worst outcomes (3 months, HR 3.59, *p* < 0.0001). The difference between *TP53/RB1*-only and *BRCA*-only groups was not significant (*p* = 0.082).

### Mutational signature profiling reveals distinct HRD-related patterns in prostate cancer

**Fig. 5:**
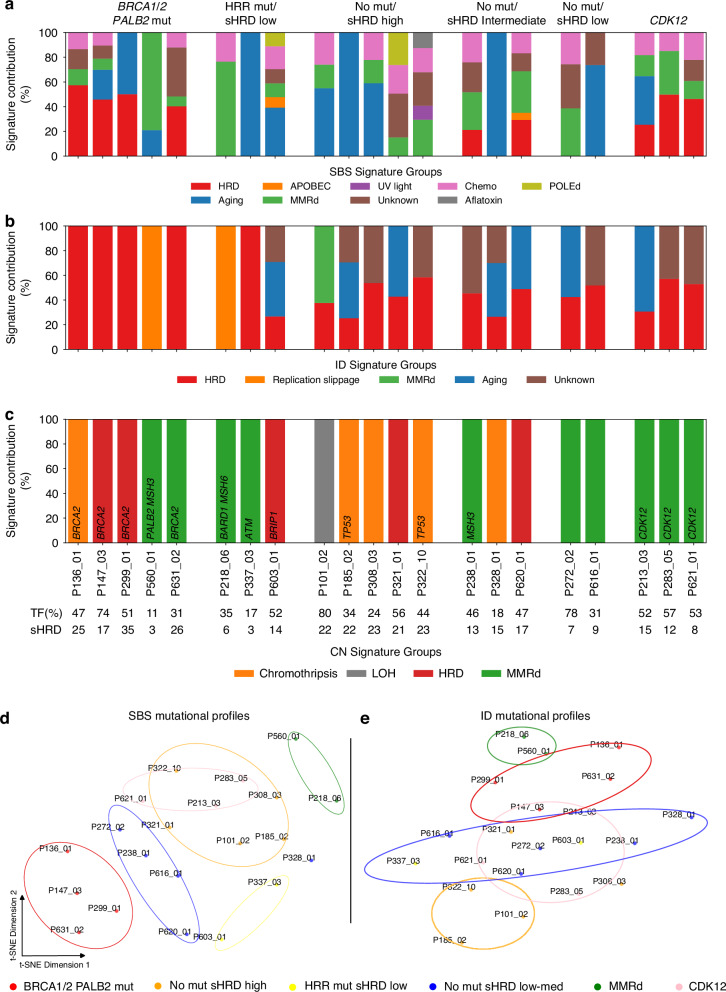
Mutational signatures reveal distinct genomic footprints of HRD, MMR deficiency, and CDK12 inactivation. **a** Single base substitution (SBS) signatures across mutation-defined and sHRD-stratified groups. **b** Indel (ID) signature composition per sample. **c** Copy number (CN) signature contributions reveal enrichment of CN25. t-SNE clustering of **d** SBS and **e** ID signatures separate HRD, *CDK12*, and MMRd cases into partially distinct groups.

Using a cfDNA optimised WES chemistry with enhanced enrichment sensitivity down to 1% VAF (Supplementary Fig. 8), we profiled mutational signatures across single base substitutions (SBS), indels (ID), doublet base substitutions (DBS), and copy number (CN) alterations to evaluate their association with HRD and to assess their potential as ctDNA biomarkers. SBS3, a hallmark HRD signature, was significantly enriched in samples with HRR mutations, including *BRCA2*- and *CDK12*-mutated tumours, and high sHRD scores, compared to those with low sHRD or no HRR mutations (*p* = 0.021). Notably, SBS3 is often absent in non-*BRCA2* tumours despite high sHRD, suggesting that sHRD alone may not fully capture HRD mutagenesis. Two borderline sHRD-high samples with detectable SBS3 levels highlight their potential as a more precise indicator (Fig. 5a).

Unexpectedly, mismatch repair deficiency (MMRd) and HRD features co-occurred in 38.1% of the cases (8/21), with MMRd-associated SBS (SBS6, 15, 26) enriched *in MSH6/MSH3*-mutated tumours (P218_06, P238_01, P560_01), of which P560_01 also had *PALB2* loss but low sHRD.

The indel signature ID6, another HRD marker, was enriched in HRR-mutated, high-sHRD samples (*p* = 0.044) but also appeared in sHRD-intermediate cases, implying HR-independent origins (Fig. 5b). ID8 was broadly detected in HRR- and *CDK12*-mutated samples but lacked discriminatory power. Among the CN signatures, CN25–linked to MMRd*—*was significantly enriched in *CDK12*-mutated tumours (*p* = 0.044 vs. HRR-mutated/high-sHRD; *p* = 0.050 vs. HRR-wildtype/high-sHRD) and tended to be higher in HRR-mutated/high-sHRD samples (*p* = 0.054), indicating potential convergence of MMRd and HRD-related instability. Classical HRD CN signatures (CN17–CN19) were present, but lacked subgroup specificity (Fig. 5c).

t-SNE clustering of raw SBS and ID calls showed a clear separation of HRD, *CDK12*, and MMRd cases, validating signature-based stratification (Fig. 5d, e). Samples without detected mutations but with high sHRD formed a distinct, partially overlapping group, emphasising the value of integrating mutational patterns with genomic instability scores for refined HRD classification.

### Fragmentomics-based detection of HRD using refined fragment features

**Fig. 6:**
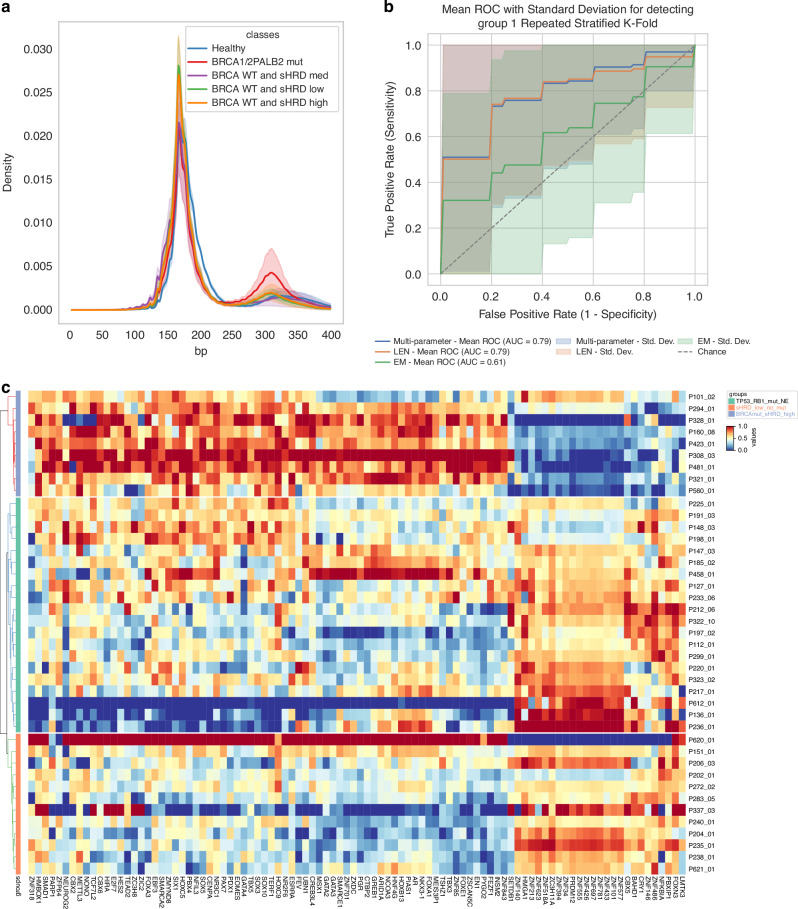
Fragmentomics and chromatin accessibility reveal non-mutational footprints of HRD in cfDNA. **a** Fragment length density plots comparing cfDNA fragmentation patterns across groups. **b** Receiver operating characteristic (ROC) curves evaluating the performance of a logistic regression classifier trained on fragment length (LEN), end motif (EM), or combined features to detect HRD-positive cases. **c** Heatmap showing differential chromatin accessibility at transcription factor binding sites (TFBS) across prostate cancer subgroups.

To investigate whether cfDNA fragmentation patterns reflect HRD, we analysed fragment length distributions, end motifs, and combined features in prostate cancer samples and healthy controls. A distinct enrichment of 290–320 bp fragments corresponding to dinucleosome lengths was observed in patients with *BRCA1/2* or *PALB2* mutations but not in other tumours or controls (Fig. 6a), suggesting altered nucleosomal organisation in HRD tumours. In contrast, end motif usage differentiated cancer from healthy samples but did not distinguish between HRD subgroups. As tumour fraction can confound cfDNA fragment size analyses, samples were grouped by mutation status and compared with *BRCA*/*PALB2*-wildtype tumours and healthy controls. Fragment counts were balanced across groups, and tumour fraction did not differ significantly between HRR-mutated and HRR-wildtype cases, indicating that neither sequencing depth nor tumour fraction biased fragment size patterns (Supplementary Fig. 11a, b).

To assess the clinical applicability of this observation, we developed a fragmentomics-based classifier for HRD detection by using penalised logistic regression. Owing to the small number of HRD-positive cases (*n* = 7), a conservative approach was used to avoid overfitting. Feature selection was performed on healthy controls only (*n* = 14) to prevent data leakage, yielding 9 fragment length and 19 end motif features. The models were evaluated using 7-fold cross-validation on 41 prostate cancer samples with high-coverage data. The fragment length-based model showed strong performance (AUC = 0.79, average precision (AP) = 0.69), whereas the end motif-only model performed modestly (AUC = 0.61, AP = 0.53). A combined model maintained the AUC (0.79) and improved the average precision (AP = 0.70), indicating added value from integrating both feature types (Fig. 6b).

### A subset of zinc finger transcription factor binding sites is less accessible in HRD cfDNA

We and others have demonstrated that transcription factor activity can be inferred from WGS data of cfDNA by analysing coverage signals at transcription factor binding sites (TFBS) [37, 41, 42]. Given that HRD is associated with widespread epigenomic alterations and chromosomal rearrangements, we hypothesised that TFBS accessibility profiles differ between HRD and HR-proficient prostate cancers.

To explore this, we conducted a differential accessibility analysis across cfDNA in a subset of our samples with available hcWGS data. Stratification based on sHRD scores did not reveal any significant differences. However, stratification into three groups by separating tumours with *TP53* and *RB1* alterations or neuroendocrine differentiation revealed distinct patterns of chromatin accessibility. These alterations are known to drive lineage plasticity, potentially overriding HRD-associated chromatin states [9], thus providing a relevant comparison. Co-mutated cases (e.g., *BRCA2* with *TP53*/*RB1*) were therefore assigned to the *TP53*/*RB1*/NE group, as combined *TP53*/*RB1* loss drives lineage plasticity, neuroendocrine transdifferentiation, and distinct chromatin profiles that could obscure HRD-specific accessibility signals. The most striking differences were observed at the zinc finger (ZNF) TFBS. In *BRCA2*-mutated, high-sHRD samples, accessibility was markedly reduced in several ZNF TFBS compared to proficient tumours (Fig. 6c, Supplementary Fig. 12). Importantly, reduced accessibility at these ZNF-binding loci was consistently observed when comparing HRD samples to both HR-proficient and NE/TP53/RB1-altered tumours; however, the signal was most pronounced in the latter group.

## Discussion

HRD has emerged as a critical predictive biomarker for PCa, guiding therapeutic decisions for PARPi and platinum-based therapies. However, reliable HRD assessment remains challenging due to tumour heterogeneity, technical difficulties of bone metastasis biopsies, and dynamic genomic evolution [43]. ctDNA analysis offers a minimally invasive alternative, but comprehensive HRD profiling via cfDNA is hindered by low TF and the complexity of GIS signatures. While previous studies have established utility of ctDNA for mutation detection [44], its use in functional, genome-wide HRD profiling has remained underexplored. We address this gap by integrating mutation data, low-pass WGS-derived copy number profiling, and mutational signatures to assess the applicability of cfDNA fragmentomics and chromatin accessibility in HRD assessment.

Consistent with prior findings [45, 46], our mutational profiling confirmed *BRCA2* as the most frequently altered HRR gene with a high proportion of germline mutations, reinforcing the importance of routine germline testing in men for familial cancer risk assessment and personalised care. Moreover, sHRD scoring from lpWGS, combined with mutation and SCNA profiles, provided a robust and cost-effective framework for assessing GIS, with SCNAs emerging as a major driver of PCa [40]. We observed biallelic inactivation in some HRR genes, though monoallelic losses were more common, potentially explaining hypomorphic HRD phenotypes. This aligns with evidence that *BRCA2* loss can cause HRD regardless of zygosity [38], although other studies suggest biallelic loss is needed for GIS or PARPi response [47, 48].

Mutational co-occurrence revealed distinct subtypes - *BRCA2* often co-occurred with *PTEN*, while *CDK12* mutations were mutually exclusive, consistent with a distinct genomic subtype. Expanding gene panels to include additional HRR and Fanconi anaemia complex genes could enhance HRD detection and therapeutic stratification [49]. Structural variant profiling revealed extensive large-scale deletions affecting HRR loci, suggesting that HRD often arises from structural loss rather than fusion events. Rare, complex SVs may also contribute to HRD phenotypes, underscoring the importance of genome-wide analyses. Tumour/normal WGS or RNA-seq could further clarify these rearrangements, which may be missed in tumour-only cfDNA assays.

The sHRD pipeline, originally designed for FFPE tissue and based on large genomic alterations (LGA ≥ 10 Mb), is a simple and efficient HRD biomarker [34]. A newer version showed good concordance with the Myriad MyChoice CDx Plus test when *BRCA1/2* status was available [50]. Its main limitation is reduced reliability at low TF, common in cfDNA assays. However, since lpWGS is low-cost and estimates TF, it enables longitudinal plasma screening to identify time points with higher TF for targeted, accurate sHRD analysis. Nonetheless, several other computational HRD classifiers have recently been developed, including HRProfiler, HRDetect, SigMA, and DARC Sign, each leveraging combinations of distinct genomic features such as HRR mutations, GIS, SCNAs, or mutational signatures [49–53]. Given the central role of mutational signatures in many HRD detection algorithms, we inferred these signatures in a subset of our cohort using a novel high-resolution WES approach, which enabled us to evaluate HRD-related mutagenesis and compare signature activity with other genomic instability metrics. In addition to elevated GIS, *BRCA2*-altered tumours showed enrichment of the SBS3 signature, whereas *TP53* and *RB1* only showed enhanced instability when combined with *BRCA2* loss. *CDK12*-mutated tumours also show SBS3 activity despite lacking classical HRD features, highlighting the complex interplay between DNA repair pathways [51]. *CDK12* alterations generate distinct forms of GIS, such as focal tandem duplications and gene fusions, that are mechanistically different from classical HRD [52]. The mutual exclusivity of *CDK12* mutations with other HRR or tumour suppressor alterations further confirms the presence of unique molecular subgroups in prostate cancer. Therefore, caution is warranted when interpreting SBS3 signatures in *CDK12*-mutant tumours, as these cases may not represent true HRD and are more likely to benefit from immune checkpoint inhibitors than PARP inhibitors [53].

Interestingly, we observed co-occurrence of signatures associated with HRD and MMR, suggesting that MMR deficiency may obscure or modify HRD phenotypes, possibly by reshaping tumour evolution or masking HR-related GIS [54]. This pattern highlights the interplay between MMR and HR, where defective MMR may influence HR activity by altering the mutational landscape and shaping tumour evolution. Given the known role of MMR in suppressing HR, its deficiency could facilitate alternative repair pathways, potentially compensating for HR defects or influencing downstream consequences [55, 56].

Clinically, high TF correlated with worse outcomes, while *TP53* and *RB1* mutations predicted poor survival, particularly with concurrent *BRCA1/2* or *PALB2* alterations. Only two patients received PARPi, potentially underestimating therapeutic benefit. These findings highlight the need for integrated risk stratification that incorporates tumour burden, GIS, and molecular subtype.

A key novelty of our study is the incorporation of cfDNA fragmentomics features to functionally characterise HRD. Fragmentation patterns, such as nucleosome phasing, fragment length distribution, and TFBS coverage, reflect chromatin accessibility and transcriptional activity in tumour-derived DNA [57]. Recent studies using integrated fragmentomics models have achieved high sensitivity for cancer detection and subtyping [58, 59]. Building on this concept, our classifier leveraged fragment length and end-motif features to capture HRD-associated fragmentation patterns in prostate cfDNA. We hypothesised that the disrupted chromatin landscape characteristic of HRD manifests as distinct cfDNA fragmentation signatures. Our findings support this view and align with recent mechanistic evidence that histone modifications and chromatin accessibility shape cfDNA fragmentation patterns, indicating that HRD-associated chromatin states can be detected through fragmentomic analysis [60].

We observed dinucleosome-length enrichment (290–320 bp) in *BRCA1/2*- and *PALB2*-mutated tumours, absent in other cases and controls, suggesting altered nucleosomal organisation. A machine learning classifier trained on fragmentomic features achieved strong cross-validated performance. Fragment length features outperformed end motifs, while their integration modestly improved precision-recall, indicating complementary biological signals. These results show that cfDNA fragmentomics captures mutation-independent structural hallmarks of HRD and may serve as a non-invasive biomarker for patient stratification in HR-targeted therapies, pending validation in independent cohorts.

To further investigate chromatin-level alterations, we analysed TFBS accessibility and identified a marked reduction in binding site accessibility for ZNF transcription factors, including ZNF34 and ZNF781, in *BRCA2*-mutant, high-sHRD tumours compared to *TP53/RB1*-mutant and neuroendocrine tumours. This epigenetic signature appears to be specific to HRD, rather than merely aggressiveness. ZNF transcription factors are among the most abundant DNA-binding proteins in the genome and are increasingly being implicated in DNA repair. For instance, ZNF281 and ZBTB24 support non-homologous end joining (NHEJ) via XRCC4 and PARP1 [61, 62], whereas PHF6 facilitates 53BP1 recruitment at DNA damage sites [63]. KDM2A and ZMYND8, with chromatin-interacting zinc finger domains, modulate histone methylation to promote RAD51 and BRCA1 recruitment during homologous recombination [64, 65]. Although ZNF34, ZNF433, and ZNF781 have not yet been directly linked to DNA repair, their consistent inaccessibility in HRD tumours suggests their roles in parallel or regulatory pathways. Downregulation or chromatin inaccessibility of ZNF genes may impair transcriptional programs essential for DNA damage recognition, chromatin remodelling, or recruitment of repair factors, ultimately contributing to a dysfunctional HR response. Furthermore, PRDM12, a histone methyltransferase, is associated with transcriptional repression via H3K9 dimethylation [66], reinforcing the connection between epigenetic dysregulation and impaired DNA repair. Overall, these findings contribute to an emerging view in which alterations in TFBS accessibility, particularly for ZNF TF, may serve as epigenetic footprints of DNA repair deficiency. This complements recent work demonstrating that many ZNF proteins are recruited to DNA damage in a PARP-dependent manner, and may act as chromatin-integrated effectors of the DSB response [67]. Future investigations should aim to clarify whether reduced accessibility at ZNF-associated sites influences specific gene regulatory networks or cofactor recruitment in HRD tumours. Whether these signatures are reversible or targetable via chromatin-modifying therapies remains an open, but promising avenue for translational research.

Although our multimodal cfDNA-based approach provides a comprehensive framework for characterising HRD in PCa, several limitations should be noted. First, clinical correlation between HRD assessment and response to PARPi or platinum-based therapies was not available, preventing direct evaluation of the predictive value of our genomic findings. Second, the limited number of confirmed HRD-positive cases, particularly those with *BRCA1/2* or *PALB2* mutations, reduces statistical power for subgroup analyses and increases the risk of overfitting in our fragmentomics classifier. However, we used a simple, predefined classifier architecture without hyperparameter tuning to avoid model overfitting, and employed repeated cross-validation to verify that performance metrics were not driven by individual samples. Third, our ground truth for HRD relied primarily on *BRCA1/2* and *PALB2* mutations identified in cfDNA since matched high-quality tissue was available for only a small subset of patients, which may underestimate functional HRD. Yet, it also underscores the need for liquid biopsy-based functional assessments of HRD, which could offer reliable, non-invasive alternatives when tumour tissue or comprehensive pathology data are unavailable. Fourth, enrichment for samples with high ctDNA fractions introduces potential selection bias and may limit generalisability to low-shedding or early-stage prostate cancer. Nonetheless, metastatic PCa typically exhibits high ctDNA burden, making it well suited for the multimodal cfDNA analysis presented here.

To extend analytical sensitivity and broaden clinical applicability, future work should explore deeper WGS, serial plasma sampling, and integration of orthogonal cfDNA-derived features (e.g., methylation, copy-number variation, nucleosome occupancy) alongside advanced normalisation and probabilistic modelling approaches. Importantly, the LOD for the proposed fragmentomic and HRD-related features remains to be systematically determined, particularly in low-ctDNA contexts, to guide their implementation in broader clinical settings. Finally, although our fragmentomics and TFBS accessibility analyses revealed novel chromatin features associated with HRD, the epigenomic signals were indirect and observational.

Despite these limitations, our findings support the feasibility of noninvasive, multimodal HRD assessment and underscore the need for standardised strategies to improve detection and therapeutic targeting in diverse patient populations. In a clinical workflow, we envision a potential classifier being integrated into a tiered HRD assessment framework, where patients first undergo targeted sequencing for HRR mutations and sHRD analysis from low-pass WGS. The fragmentomics classifier in combination with chromatin accessibility features could then serve as a secondary or complementary tool to refine HRD classification, particularly in cases with ambiguous or discordant molecular results. Independent validation and prospective clinical trials will be required to validate this framework and assess its feasibility in real-world settings.

## Supplementary information

Supplementary

## Data Availability

The data supporting the findings of these studies are available from the corresponding authors upon request. All raw sequencing data were deposited in the European Genome-phenome Archive (EGA; http://www.ebi.ac.uk/ega/), hosted by the EBI, under the accession number EGAS00001008190. Processed data and original code used in analyses are available at https://github.com/vlachosg37/HRD_ctDNA_publication_release.
