# Supplementary material for: Functional footprints of homologous recombination deficiency in prostate cancer revealed by ctDNA fragmentation and transcription factor accessibility
Source: Br J Cancer. 2026 Jan 9;134(6):949–60. doi: 10.1038/s41416-025-03301-0 (PMC12960696; doi:10.1038/s41416-025-03301-0)
Supplement: Supplementary file 1 — Supplementary [file 41416_2025_3301_MOESM1_ESM.docx]

**Supplementary material**

**Functional Footprints of Homologous Recombination Deficiency in Prostate Cancer Revealed by ctDNA Fragmentation and Transcription Factor Accessibility**

**Running title: Fragmentomics Uncovers Non-Mutational HRD Features**

Georgios Vlachos^1,2^, Tina Moser^1^, Isaac Lazzeri^1,2^, Matthias J. Moser^1^, Lisa Glawitch^1^, Emil Thomas Bauernhofer^3^, Anna Eberhard^1^, Christine Beichler^1^, Hanieh Sadeghi^1^, Jasmin Blatterer^1^, Stefan Kühberger^1^, Nina Monsberger^1^, Karl Kashofer^4^, Jochen B. Geigl^1^, Thomas Bauernhofer^3^, Ellen Heitzer^1,2^

^1^Institute of Human Genetics, Diagnostic & Research Center for Molecular BioMedicine, Medical University of Graz, Austria, ^2^Christian Doppler Laboratory for Liquid Biopsies for Early Detection of Cancer, Medical University of Graz, Neue Stiftingtalstrasse 6, 8010 Graz, Austria, ^3^Department of Internal Medicine Graz, Division of Oncology, Medical University of Graz, ^4^Diagnostic and Research Institute of Pathology, Medical University Graz, Graz, Austria

Table of Contents

[1 Supplementary Methods 3](#_Toc212073425)

[1.1 Sample collection and cfDNA isolation 3](#_Toc212073426)

[1.2 Modified Fast Aneuploidy Screening Test-Sequencing System (mFAST-SeqS) 4](#_Toc212073427)

[1.3 Panel analysis of clinically relevant genes 4](#_Toc212073428)

[1.4 Co-occurrence analysis 5](#_Toc212073429)

[1.5 WES library preparation and sequencing 5](#_Toc212073430)

[1.6 Mutational signature analysis 6](#_Toc212073431)

[1.7 Whole genome sequencing 6](#_Toc212073432)

[1.8 Fragment feature analysis 7](#_Toc212073433)

[1.9 Transcription factor binding analysis 8](#_Toc212073434)

[1.10 Statistical analysis 9](#_Toc212073435)

[2 Supplementary Tables 9](#_Toc212073436)

[2.1 Supplementary Table 1. Patient characteristics and clinical data 9](#_Toc212073437)

[2.2 Supplementary Table 2. Performance assessment of the QIASeq amplicon panel 10](#_Toc212073438)

[2.3 Supplementary Table 3. Co-occurrence matrix of pathogenic mutations 11](#_Toc212073439)

[3 Supplementary Figures 12](#_Toc212073440)

[Supplementary Figure 1. Summary of tumor fractions assessed by mFAST-SeqS and lpWGS. 12](#_Toc212073441)

[Supplementary Figure 2. Kaplan Meier plots stratifying the patients by PCa subtype. 13](#_Toc212073442)

[Supplementary Figure 3. Correlation between tumor fraction, variant allele frequency, and genomic instability scores in plasma cfDNA. 14](#_Toc212073443)

[Supplementary Figure 4. Somatic landscape and mutation distribution of HRD-associated genes in plasma cfDNA. 15](#_Toc212073444)

[Supplementary Figure 5. Genome-wide copy number alteration frequencies across cfDNA prostate cancer subgroups. 16](#_Toc212073445)

[Supplementary Figure 6. Association between sHRD score and genomic alterations. 17](#_Toc212073446)

[Supplementary Figure 7. Kaplan–Meier survival analysis stratified by genomic instability (sHRD score). 18](#_Toc212073447)

[Supplementary Figure 8. Detection sensitivity and variant allele frequencies in a reference standard across VAF thresholds. 19](#_Toc212073448)

[Supplementary Figure 9. Fragmentomic profiles stratified by HRD mutation status and genomic instability. 20](#_Toc212073449)

[Supplementary Figure 10. Somatic landscape of Fanconi anemia (FANC) pathway genes in prostate cancer WES data. 21](#_Toc212073450)

[Supplementary Figure 11. Fragment length distributions stratified by HRR mutation status. 22](#_Toc212073451)

[Supplementary Figure 12. Quantification of differential accessibility transcription factor binding sites. 23](#_Toc212073452)

[Supplementary Figure 13. Structural variant landscape in cfDNA WGS samples. 24](#_Toc212073453)

# Supplementary Methods

## Sample collection and cfDNA isolation

In total, 898 plasma samples were collected from 375 patients diagnosed with metastatic prostate cancer between 2012 and 2023. Blood samples were collected in Cell-Free DNA blood collection tubes (Streck) or PAXgene Blood ccfDNA Tubes (PreAnalytiX) at the Division of Oncology at the Medical University of Graz, Austria. The study protocol and informed consent forms were approved by the local ethics committee (EK Nr ex 21-228). Written informed consent was obtained from all participants for collection of blood specimens used for plasma genotyping and white blood cell collection for germline testing. Plasma was extracted as previously described using a double spin at 1900 × g for 10 min (1). Buffy coat was collected after the first spin. Plasma and white blood cells were stored at -80°C until DNA extraction. cfDNA was extracted from 2 ml of plasma using the QIAamp Circulating Nucleic Acid Kit (QIAGEN, Hilden, Germany) according to the manufacturer’s instructions and quantified using the Qubit dsDNA HS Assay Kit on a Qubit 4 Fluorometer (RRID: SCR_026883, Invitrogen, ThermoFisher Scientific, USA).

Each plasma sample was treated as an independent biological replicate, representing a single patient and time point. No technical replicates were used, as all sequencing libraries were independently prepared and processed. Sample grouping was based on molecular and clinical characteristics, and neither randomization nor blinding was applied. Data analyses were performed retrospectively after molecular processing, with group allocation (e.g., HRD status) determined by objective genomic criteria.

## Modified Fast Aneuploidy Screening Test-Sequencing System (mFAST-SeqS)

To estimate aneuploidy and tumor fraction, mFAST-SeqS was used, as previously described (2). This method detects copy number changes across the genome by amplifying and sequencing repetitive LINE-1 elements that are evenly distributed across chromosomes. Briefly, 1 ng of cfDNA was amplified with LINE1-specific primers (five PCR cycles), purified with AMPure beads (Beckman Coulter, Brea, CA, USA), and further amplified by 18-cycle PCR with Illumina adapters and indices. After purification and quantification, libraries were sequenced on the Illumina platform in paired-end mode aiming for 100k reads. Reads were aligned to hg19/GRCh37 using BWA-MEM2 (RRID: SCR_022192, v.0.7.4) (3), and those with a mapping quality >15 were counted per chromosome arm. Z-scores were calculated by normalizing read counts against those of 35 healthy controls to assess chromosomal arm-level aneuploidy. A genome-wide z-score was derived by summing the squared normalized values across all chromosome arms.

From this analysis, 106 plasma samples were identified and proceeded to targeted sequencing for detailed characterization of HRR gene alterations. As this was an observational exploratory study designed to characterize HRD-associated cfDNA features rather than to test a predefined effect size, no formal power calculation was performed. The final cohort size (n = 106) was determined by the availability of high-quality plasma samples meeting sequencing and tumor fraction criteria.

## Panel analysis of clinically relevant genes

Mutations in HRR genes were assessed using the QIAseq HRR panel (Qiagen, Hilden, Germany) targeting *ATM, BARD1, BRCA1, BRCA2, BRIP1, CDK12, CHEK1, CHEK2, FANCL, PALB2, PPP2R2A, RAD51B, RAD51C, RAD51D*, and *RAD54L*. Additionally, we customized the panel by including primers covering the entire coding region of *RB1, PTEN,* and *TP53*. Assay performance was evaluated using Seraseq ctDNA Complete™ Mutation Mix standards (Seracare, MA, USA) at VAFs of 5%, 2.5%, 1%, 0.5%, 0.1%, and wild-type, and the extended panel was validated with the ctDNA™ Mutation Mix v2 (1%, 0.5%, 0.125% VAFs, and WT), mimicking plasma conditions across the entire workflow. Libraries were prepared from 10 to 40 ng of cfDNA, according to the manufacturer’s recommendations. The DNA was enzymatically fragmented and ligated with adapters containing Unique Molecular Identifiers (UMIs) and indices. Target regions were enriched via an 8-cycle PCR followed by 20-cycle universal PCR. Library quality and size were assessed using an Agilent 2100 Bioanalyzer (RRID: SCR_018043), and quantification was performed with StepOne qPCR (RRID: SCR_023455) using the QIAseq Library Quant Assay (Qiagen, Hilden, Germany). Libraries were pooled equimolarly and sequenced in 2 × 150 paired-end mode on an Illumina NextSeq 550 (RRID: SCR_016384) or NovaSeq 6000 (RRID: SCR_016387), aiming for ≥25,600× coverage to reliably detect variants at 1% VAF.

FASTQ files from targeted panel sequencing were processed on the GeneGlobe platform (RRID: SCR_021211, QIAGEN), which included adapter trimming and filtering of short reads. The reads were aligned to the hg19/GRCh37 reference genome using BWA-MEM2 (RRID: SCR_022192, v0.7.9a-r786). Unique Molecular Identifiers (UMIs) were clustered before variant calling using **smCounter2** (4). Variant annotation was performed using **Golden Helix VarSeq (RRID: SCR_001285, v2.2.0),** Golden Helix Inc., Bozeman, USA) with functional predictions based on **gnomAD (RRID: SCR_014964)** and **dbNSFP (RRID: SCR_005178)** (5,6). Variants were classified into five categories according to **ACMG-AMP (RRID: SCR_005769) guidelines** (7): benign, likely benign, VUS, likely pathogenic, and pathogenic. Manual curation was performed for variants with VAFs <0.5% as low-frequency mutations were reliably detected during assay validation.

## Co-occurrence analysis

The calculation of expected co-occurrence is based on the assumption that two alterations occur independently. For each alteration A, the probability P(A) was calculated by dividing the number of patients with the alteration by the total number of patients. The expected number of co-occurrences between alterations A and B is the product of their individual probabilities multiplied by the Total Number of Patients expected to co-occur (A, B) = P(A) × P(B) × Total Number of Patients (8).

## WES library preparation and sequencing

Whole-exome sequencing was performed on 21 cfDNA tumor samples and matched germline DNA from peripheral blood using a tumor-normal design. For each tumor sample, 20 ng of cfDNA was used as input for library preparation using the Illumina DNA Prep with Exome 2.5 Enrichment kit, which includes the Twist Exome 2.5 panel. Germline libraries were prepared using Twist Human Exome 2.0. Libraries were quantified using the Qubit™ dsDNA HS Assay and StepOne qPCR (RRID: SCR_023455), and fragment sizes were assessed using Agilent 7500 kits. Plasma libraries were sequenced in three NovaSeq 6000 S4 flow cells (eight samples per flow cell), while germline samples were sequenced on a single S1 flow cell. All sequencing was performed in 2×150 bp paired-end mode. The average coverage for the tumor libraries exceeded 1500×, and the two reference standards used had >1600× coverage. The germline coverage ranged from 28× to 288× (mean: 150×, median: 178×).

Variant calling sensitivity was evaluated using the Twist cfDNA Pan-Cancer Reference Standard, v2. Of the 151 expected clinically relevant mutations, 100% of the 5% VAF variants and 94.7% of the 1% VAF variants were detected **(*Supplementary Figure 8*).**

## Mutational signature analysis

Mutational signatures for SBS, DBS, and ID were called using the MutationalPatterns (RRID: SCR_024247) package with the strict refit method, utilizing COSMIC (RRID: SCR_002260, v3.4) for signature refitting (9,10). Alignment of FASTQ files was performed with BWA-MEM2 (RRID: SCR_022192, v0.7.9a-r786) (3) on the hg38 reference genome, and variants were called using Mutect2 (RRID: SCR_026692), following GATK (RRID: SCR_001876) best practices. CN signatures were called using SigProfilerExtractor with the VCF files generated from variant calling and allele-specific copy number alterations identified using Sequenza (RRID: SCR_016662) (11). Data normality was assessed using the Shapiro-Wilk test. Two-sided Analysis of variance (ANOVA) and two-sided Mann-Whitney U tests were used to compare signature contributions across groups. Statistical significance was defined as p<0.05, and analysis was performed using Python (RRID: SCR_008394, 3.11.7)

## Whole genome sequencing

WGS libraries were prepared from 10 ng of cfDNA using the TruSeq DNA Nano Sample Preparation Kit (Illumina, San Diego, CA, USA) as described previously (12). Libraries were quantified using the Qubit™ dsDNA High Sensitivity Assay Kit (Invitrogen, ThermoFisher Scientific, USA) and quality-checked using the Agilent DNA 7500 Kit (Agilent, Santa Clara, USA). Pooled libraries were quantified by qPCR using the StepOne qPCR System (RRID: SCR_023455). For lpWGS, 106 libraries were pooled equimolarly and sequenced on a NovaSeq 6000 system (RRID: SCR_016387) using an SP flow cell, generating approximately 0.1–0.2× coverage per sample. High-coverage WGS (hcWGS) was performed on 42 cfDNA samples to enable comprehensive genomic analysis, including mutational signature calling and copy number profiling. These libraries were sequenced on a NovaSeq 6000 S4 flow cell in 2×101 bp mode, achieving an average coverage of ~30× per sample.

The aligned BAM files were generated using the hg19 reference genome. PCR duplicates were identified and marked using Picard’s MarkDuplicates (RRID: SCR_006525) (13). Reads failing quality control, such as unmapped reads, secondary or supplementary alignments, and those with mapping quality <20 were excluded from downstream analysis. Copy number alterations (CNAs) were called using ichorCNA (RRID: SCR_024768) on lpWGS data (∼0.1–0.2 × coverage) (14). A custom script was used to aggregate the segments of 106 patient samples. To retrieve heterozygous events, CNA events with an absolute median log2 ratio lower than half of the tumor fraction of the respective sample were filtered out. Events of copy number gain (GAIN, AMP, HLAMP, HLAMP2, and HLAMP3) were compiled into AMP to simplify the analysis. Calls with fewer than five bins were filtered. Gene loci were retrieved from the USCS Genome Browser Gateway (15) using human assembly hg19. The events were then matched with the loci of the genes if the start of the altered segment was upstream of the gene locus and the end of the segment downstream of the gene locus.

Genomic instability was assessed using the shallowHRD tool (16). The analysis was performed in R (RRID: SCR_001905, v.4.3.3) using the published shallowHRD script, which infers HRD status based on the number of large-scale genomic alterations (LGAs) detected from segmented copy number profiles at low coverage. The samples were classified as HRD-positive or non-HRD using established LGA thresholds, as described in the original publication.

Structural variants (SVs) were detected from high-coverage WGS data using Manta v1.6.0 (RRID:SCR_022997) (Illumina, San Diego, CA) in tumor-only mode (17). The hg38 reference genome was used for alignment and variant calling. Resulting VCF files were filtered to retain high-confidence events ≥50 bp, including deletions, duplications, inversions, and translocations. SVs were annotated with the Ensembl Variant Effect Predictor (RRID:SCR_007931, VEP v115) in offline mode using the corresponding Ensembl cache release 115 and the --everything flag for comprehensive functional annotation. Annotated variants were subsequently filtered for events overlapping genes in the homologous recombination repair (HRR) pathway (Supplementary Table 2) (18).

## Fragment feature analysis

After alignment to the hg38 reference genome, PCR duplicates were marked using Picard’s MarkDuplicates (RRID: SCR_006525) (3,13) and removed along with unmapped reads, low mapping quality reads (MAPQ < 20), secondary, and supplementary alignments. Fragment lengths were extracted directly from the filtered BAM files. For end-motif analysis, the 4-nucleotide (4-mer) sequence at the 5′ end of each cfDNA fragment was extracted and used as the basis for feature construction.

Fragment length analysis was limited to cfDNA fragments of 80–400 base pairs (bp). For each sample, fragment length counts were converted to relative frequencies and log10-transformed. A 32 bp rolling average was applied to smooth the frequency distribution, and the resulting values were Z-score standardized using the mean and standard deviation from an independent cohort of 14 healthy gender- and age-matched control samples. The smoothed z-score distributions were then divided into nine non-overlapping 32 bp bins. The final set of fragment length features consisted of the standard deviation of Z-scores within each bin.

The initial set of 256 possible 4-mer end motifs was normalized using Centered Log-Ratio (CLR) transformation. Spectral Clustering was then applied to a target of 25 clusters. To reduce noise from infrequent motifs, clusters composed primarily of low-frequency motifs (mean frequency < 0.008) were excluded following visual inspection, resulting in a refined feature set of 19 representative end-motif clusters.

Owing to the small number of HRD-positive samples, conventional training/test splits were avoided to reduce the variance. Instead, model performance was evaluated using a 100-fold repeated stratified cross-validation. In each iteration, a penalized logistic regression model (L2 regularization, C = 1) is trained on the selected length and motif features. A late fusion strategy was applied by averaging the positive class probabilities across all the 100 models to generate the final prediction scores per sample. This approach allowed for stable estimates and mitigated overfitting owing to class imbalance and sample-size constraints.

To address the inherent challenges of a small sample size and class imbalance, we adopted a modeling strategy specifically designed for rare-event detection. Features were engineered using healthy control data alone to prevent data leakage and to capture biologically grounded deviations in cfDNA fragmentation. Rather than relying on aggregate metrics, we extracted localized patterns in fragment length variability and clustered end-motif sequences to retain interpretable structures while reducing noise. The classifier performance was evaluated using repeated stratified cross-validation with model averaging, providing stable and generalizable estimates despite the limited number of HRD-positive cases. This approach balances robustness with interpretability and supports the reliable discrimination of HRD status from subtle cfDNA fragmentomics signals.

## Transcription factor binding analysis

To investigate chromatin accessibility at TFBS in cfDNA, we applied the LBFextract framework (v0.1.0a1) for feature extraction and differential analysis (19). LBFextract is a modular Python-based package designed specifically for WGS data from liquid biopsies and enables systematic quantification of chromatin-associated signals around predefined genomic features.

Coverage signals were calculated at TFBS loci defined in BED format using fragment coverage derived from paired-end WGS cfDNA alignments. The signal at each site was summarized using a normalized mean coverage approach across a 4,000 bp window (±2,000 bp from the TFBS center), and flanking regions were used to adjust for global depth variability. To correct for GC bias at the fragment level, we used GCparagon, a tool specifically designed to address fragment length–dependent GC biases in cfDNA WGS data while enabling rapid computation. GCparagon assigns GC bias weights to individual reads, which are incorporated by LBFextract during coverage calculation, ensuring accurate GC-corrected fragment coverage across samples (20).

For transcription factor specific analysis, TFBS data were retrieved from the GTRD database (version 21.12). Specifically, we downloaded the Homo_sapiens_meta_clusters.zip file and split the data by the tfTitle column, ranking the entries by peak.count - the number of ChIP-seq experiments supporting each metacluster. We then excluded all TFBS regions (±2,000 bp around each TFBS center) that overlapped with the ENCODE Blacklist V2 (hg38) to eliminate problematic genomic regions. From the remaining TFBSs, we selected the top 1,000 sites per TF, ranked by peak.count, ensuring high-confidence binding sites. Coverage extraction was performed for all 1,048 TFs with at least 1,000 TFBSs after blacklist filtering using LBFextract. The resulting coverage signals were normalized in multiple steps:

1. *In silico* normalization was applied to achieve consistent tumor fraction and sequencing coverage across samples, following the procedure described by Lazzeri et al. (19). Specifically, patient-derived reads were computationally mixed with randomly drawn reads from a pool of healthy donor cfDNA to generate synthetic samples with standardized tumor fraction and coverage.
2. Local signal normalization was performed within LBFextract, which applies mean signal normalization using the flanking regions (−2000 to −1000 bp and +1000 to +2000 bp from each TFBS center) to control for regional variation in read depth and coverage bias.
3. For differential TF accessibility analysis, accessibility scores were extracted for each TF across all samples, and outliers were removed. Group comparisons (e.g., HRD vs. HRR-proficient) were performed using the Mann–Whitney U test, and the resulting p-values were corrected for multiple testing using the Benjamini–Hochberg false discovery rate (FDR) procedure. Transcription factors with an adjusted p < 0.05 were considered significantly differentially accessible.

## Statistical analysis

All statistical analyses were performed in Python (RRID: SCR_008394, v3.11.7) and R (RRID: SCR_001905, v4.3.3). Data normality was assessed using the Shapiro–Wilk test. For normally distributed data, approximate equality of variances between groups was verified by inspection of residuals. For normally distributed data with homogeneous variances, two-sided parametric tests (t-test or ANOVA) were applied; otherwise, two-sided non-parametric tests (Mann–Whitney U) were used. Correlations were assessed using two-sided Spearman tests, and multiple comparisons were adjusted using the Benjamini–Hochberg false-discovery-rate (FDR) method. Statistical tests were selected and justified based on data type and distribution, and all test names, p-values, and effect sizes (e.g., hazard ratios with 95% CI) are reported in the figure legends and corresponding Methods sections. Center values are reported as median, and measures of variability are provided when appropriate: mean ± standard deviation (s.d.) for normally distributed data or interquartile range (IQR) for non-parametric data. All statistical tests were two-sided, and p < 0.05 was considered statistically significant.

# Supplementary Tables

## Supplementary Table 1. Patient characteristics and clinical data

| **Patient Characteristic** | **CRPC (n=60)** | **HSPC (n=33)** | **Unknown (n=6)** | **nePC (n=7)** | **Total (n=106)** |
| --- | --- | --- | --- | --- | --- |
| **Age** | 63.0 (47.0–88.0) | 68.0 (55.0–84.0) | 73.0 (66.0–80.0) | 62.0 (47.0–65.0) | 65.0 (47.0–88.0) |
| **Gleason score** | 7 (6-9) | 8 (6-8) | 7 (6-8) | 8 (8-8) | 7 (6-9) |
| **Metastatic diagnosis** | Metachronous: 34 | Metachronous: 10 | NA | Metachronous: 3 | Metachronous: 47 |
|  | De novo: 24 | De novo: 20 | NA | De novo: 4 | De novo: 48 |
|  | No: 2 | No: 3 | NA | NA | No: 5 |
| **Radiotherapy as primary treatment** | Yes: 14 | Yes: 7 | Yes: 0 | Yes: 0 | Yes: 21 |
|  | No: 46 | No: 26 | No: 6 | No: 7 | No: 85 |
|  | NA | NA | NA | NA | NA |
| **Palliative radiotherapy** | Yes: 34 | Yes: 19 | Yes: 0 | Yes: 5 | Yes: 58 |
|  | No: 24 | No: 14 | No: 2 | No: 2 | No: 42 |
|  | NA: 2 | NA | NA: 4 | NA | NA: 6 |
| **Neoadjuvant hormone therapy** | Yes: 12 | Yes: 2 | Yes: 1 | Yes: 0 | Yes: 15 |
|  | No: 48 | No: 31 | No: 1 | No: 7 | No: 87 |
|  | NA | NA | NA:4 | NA | NA: 4 |
| **PSA doubling time (Months)** | 2.2 (0.25–25.3) | 1.85 (1.1–13.7) | NA | 1.8 (1.1–3.8) | 2.05 (0.25–25.3) |
| **Karnofsky performance index** | 80.0 (50.0–100.0) | 80.0 (50.0–100.0) | NA | 90.0 (60.0–100.0) | 80.0 (50.0–100.0) |
| **Alkaline phosphatase (U/L)** | 221.0 (44.0–1721.0) | 131.0 (63.0–1626.0) | 582.5 (534.0–631.0) | 130.0 (69–272) | 206.0 (44.0–1721.0) |
| **Lactate dehydrogenase (U/L)** | 309.0 (165.0–2411.0) | 246.5 (163.0–1106.0) | 584.0 (584.0–584.0) | 657.0 (227–1789) | 292.0 (163.0–2411.0) |
| **Hemoglobin (g/L)** | 11.55 (7.5–15.4) | 12.65 (9.1–15.8) | 10.8 (10.8–10.8) | 10.8 (8.8–13.5) | 11.7 (7.5–15.8) |
| **PSA (ng/mL, plasma)** | 162.35 (0.01–10000.0) | 16.89 (0.01–2397.34) | 229.0 (4.61–453.39) | 22.75 (3.2–204.51) | 55.52 (0.01–10000.0) |
| **ctDNA fraction** | 31.35 (0.0–80.34) | 20.67 (0.0–71.05) | 21.73 (5.9–74.92) | 47.53 (7.35–63.6) | 30.32 (0.0–80.34) |
| **sHRD score** | 13.0 (0–30) | 5.0 (0–24) | 18.5 (0–35) | 10.0 (2–29) | 11.0 (0–35) |
| **Z-score** | 36.48 (1.8–350.87) | 11.43 (2.24–376.65) | 23.43 (2.98–49.06) | 53.51 (31.33–210.35) | 33.93 (1.8–376.65) |
| **Follow-up for OS (months)** | 76.5 (4.0–255.0) | 56.0 (2.0–237.0) | 16.5 (2.0–31.0) | 52.0 (20.0–192.0) | 61.5 (2.0–255.0) |

## Supplementary Table 2. Performance assessment of the QIASeq amplicon panel

| **Gene** | **HGVS c** | **HGVS p.** | **WT** | **.125% VAF** | **.5% VAF** | **1% VAF** |
| --- | --- | --- | --- | --- | --- | --- |
| **ATM** | c.1058_1059delGT | p.Cys353Serfs*5 | ND | ND | 0.56% | 1.10% |
| **PTEN** | c.741dupA | p.Pro248Thrfs*5 | ND | ND | ND | 0.76% |
| **PTEN** | c.800delA | p.Lys267Argfs*9 | ND | ND | ND | 0.75% |
| **TP53** | c.818G>A | p.Arg273His | ND | ND | 0.68% | 1.12% |
| **TP53** | c.743G>A | p.Arg248Gln | ND | ND | 0.50% | 0.90% |
| **TP53** | c.723delC | p.Cys242Alafs*5 | ND | ND | 0.51% | 0.85% |
| **TP53** | c.524G>A | p.Arg175His | ND | ND | 0.60% | 1.30% |
| **TP53** | c.267delC | p.Ser90Profs*33 | ND | ND | 0.46% | 1.40% |

## Supplementary Table 3. Co-occurrence matrix of pathogenic mutations

|  | ***RB1*** | ***TP53*** | ***BRCA2*** | ***CHEK2*** | ***ATM*** | ***PTEN*** | ***CDK12*** | ***CHEK1*** | ***BRCA1*** | ***PALB2*** | ***BRIP1*** | ***RAD54L*** | ***RAD51B*** | ***FANCL*** | ***BARD1*** |
| --- | --- | --- | --- | --- | --- | --- | --- | --- | --- | --- | --- | --- | --- | --- | --- |
| ***RB1*** | 0 | 0.02 | 0.11 | 0 | 0 | 0.05 | 0 | 0 | 0 | 0 | 0 | 0 | 0 | 0 | 0 |
| ***TP53*** | 0.02 | 0 | 0.05 | 0.06 | 0 | 0.1 | 0 | 0 | 0.03 | 0.03 | 0 | 0 | 0 | 0 | 0.03 |
| ***BRCA2*** | 0.11 | 0.05 | 0 | 0 | 0 | 0.19 | 0 | 0 | 0 | 0 | 0 | 0 | 0 | 0 | 0 |
| ***CHEK2*** | 0 | 0.06 | 0 | 0 | 0 | 0.09 | 0 | 0 | 0 | 0 | 0 | 0 | 0 | 0 | 0 |
| ***ATM*** | 0 | 0 | 0 | 0 | 0 | 0 | 0 | 0 | 0.25 | 0 | 0 | 0 | 0 | 0 | 0 |
| ***PTEN*** | 0.05 | 0.1 | 0.19 | 0.09 | 0 | 0 | 0 | 0 | 0 | 0 | 0 | 0 | 0 | 0 | 0.1 |
| ***CDK12*** | 0 | 0 | 0 | 0 | 0 | 0 | 0 | 0 | 0 | 0 | 0 | 0 | 0 | 0 | 0 |
| ***CHEK1*** | 0 | 0 | 0 | 0 | 0 | 0 | 0 | 0 | 0 | 0 | 0 | 0 | 0 | 0 | 0 |
| ***BRCA1*** | 0 | 0.03 | 0 | 0 | 0.25 | 0 | 0 | 0 | 0 | 0 | 0 | 0 | 0 | 0 | 0 |
| ***PALB2*** | 0 | 0.03 | 0 | 0 | 0 | 0 | 0 | 0 | 0 | 0 | 0 | 0 | 0 | 0 | 0 |
| ***BRIP1*** | 0 | 0 | 0 | 0 | 0 | 0 | 0 | 0 | 0 | 0 | 0 | 0 | 0 | 0 | 0 |
| ***RAD54L*** | 0 | 0 | 0 | 0 | 0 | 0 | 0 | 0 | 0 | 0 | 0 | 0 | 0 | 0 | 0 |
| ***RAD51B*** | 0 | 0 | 0 | 0 | 0 | 0 | 0 | 0 | 0 | 0 | 0 | 0 | 0 | 0 | 0 |
| ***FANCL*** | 0 | 0 | 0 | 0 | 0 | 0 | 0 | 0 | 0 | 0 | 0 | 0 | 0 | 0 | 0 |
| ***BARD1*** | 0 | 0.03 | 0 | 0 | 0 | 0.1 | 0 | 0 | 0 | 0 | 0 | 0 | 0 | 0 | 0 |

# Supplementary Figures


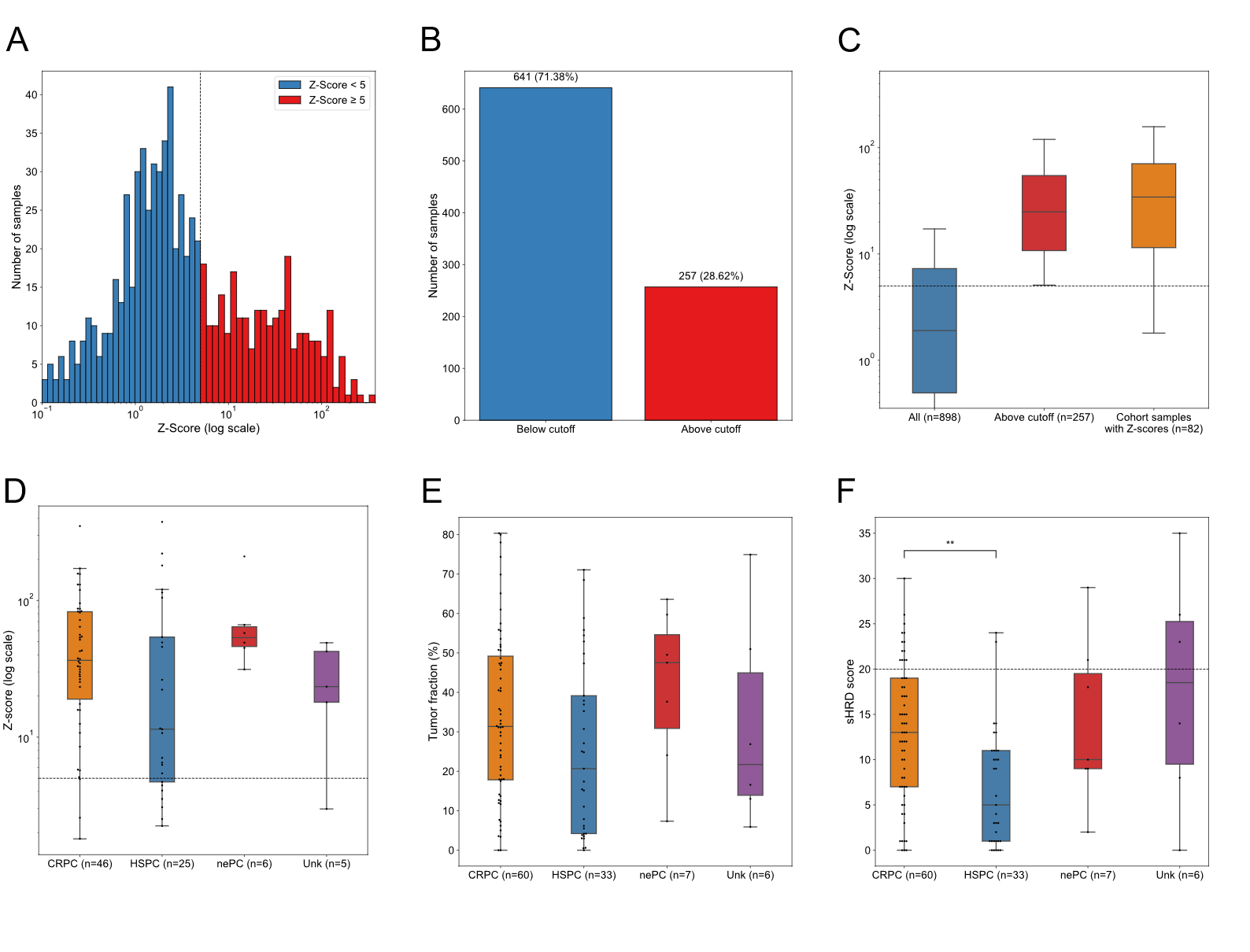
Supplementary Figure 1. Summary of tumor fractions assessed by mFAST-SeqS and lpWGS. **A)** Histogram of Z-scores derived from mFAST-SeqS across 898 plasma samples from 375 prostate cancer patients. A threshold of Z > 5 was used to enrich for samples with estimated TF ≥ 5–10%. **B)** Proportion of samples above and below the Z-score cutoff. **C)** Boxplot of Z-scores (log scale) in all samples, those above the cutoff, and the final high-TF subset used for genomic analysis. **D)** Z-score distribution across four clinical subgroups: castration-resistant prostate cancer (CRPC), hormone-sensitive prostate cancer (HSPC), neuroendocrine prostate cancer (nePC), and unclassified (Unk). **E)** Tumor fraction (TF) distribution across across the same subgroups. **F)** sHRD scores across the same subgroups. A significant difference in sHRD was observed between mCRPC and mHSPC groups (p < 0.001, two-sided Mann–Whitney U test). In all boxplots, the center line denotes the median, box edges indicate the interquartile range (IQR), and whiskers extend to 1.5× IQR. Individual data points are overlaid as black dots.

***
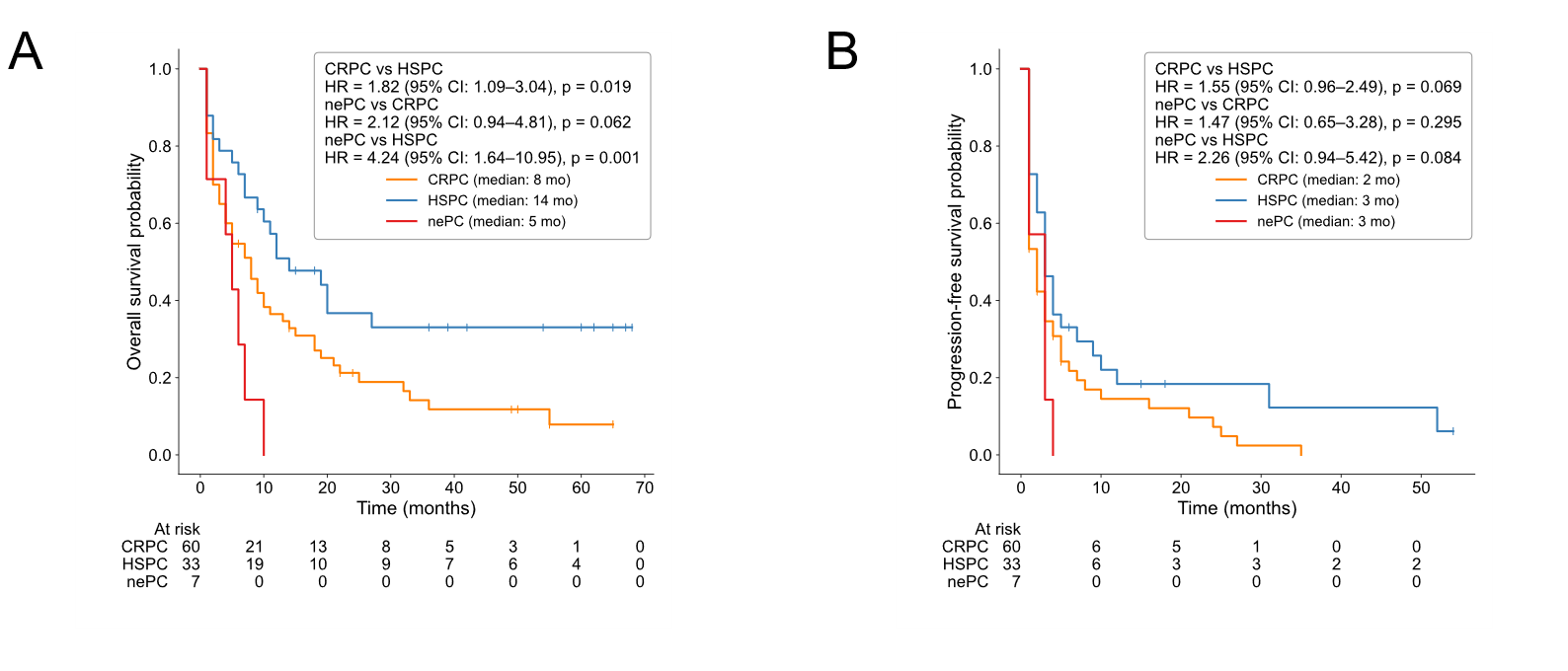
***

Supplementary Figure 2. Kaplan Meier plots stratifying the patients by PCa subtype. **A)** Overall survival (OS) and **B)** progression-free survival (PFS) stratified by clinical group: castration-resistant prostate cancer (CRPC), hormone-sensitive prostate cancer (HSPC), and treatment-emergent neuroendocrine prostate cancer (nePC). Median survival times are indicated in the legend.
Hazard ratios (HR), 95% confidence intervals (CI), and log-rank p-values are shown for pairwise comparisons between groups. Censoring is indicated by vertical tick marks. At-risk tables are shown below each plot. Survival curves were estimated using the Kaplan–Meier method; HRs were derived from Cox proportional hazards models.


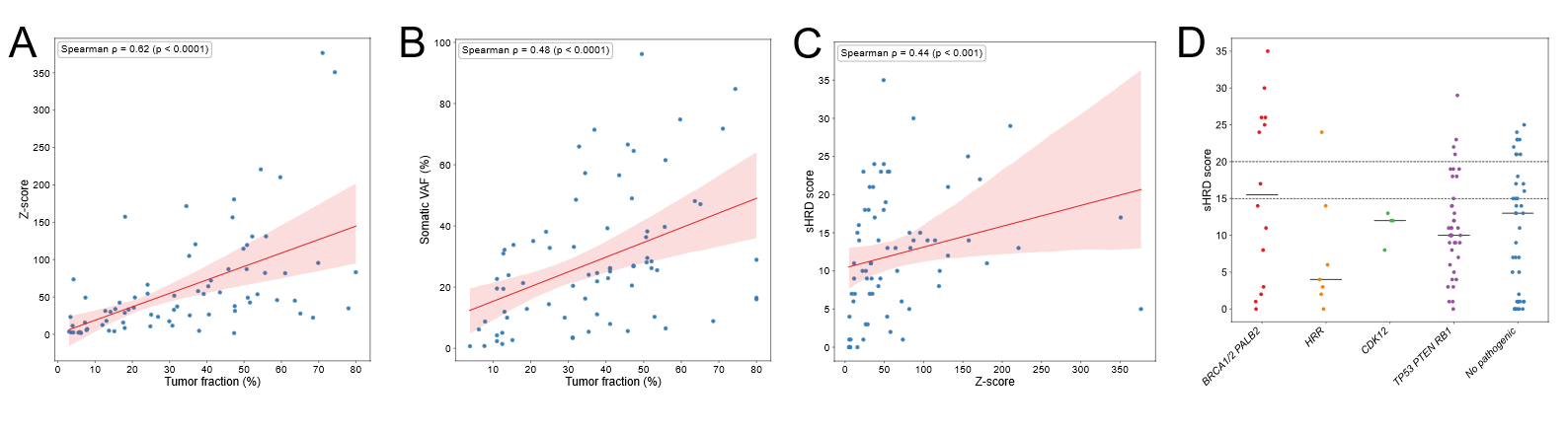
Supplementary Figure 3. Correlation between tumor fraction, variant allele frequency, and genomic instability scores in plasma cfDNA. **A)** Scatterplot comparing tumor fraction (TF) estimated by lpWGS with Z-scores. **B)** Scatterplot of TF versus the median variant allele frequency (VAF) of somatic mutations detected in each sample. **C)** Scatterplot comparing Z-scores with corresponding sHRD scores derived from lpWGS, reflecting large-scale genomic instability. Spearman correlation coefficients and p-values are shown in panels A-C. **D)** Strip plot showing the distribution of sHRD scores across five mutation-defined subgroups: *BRCA1/2* or *PALB2*, other HRR genes, *CDK12*, *TP53/PTEN/RB1*, and samples without pathogenic mutations. Horizontal dashed lines indicate sHRD thresholds of 15 and 20. Median values for each group are indicated with horizontal black lines.


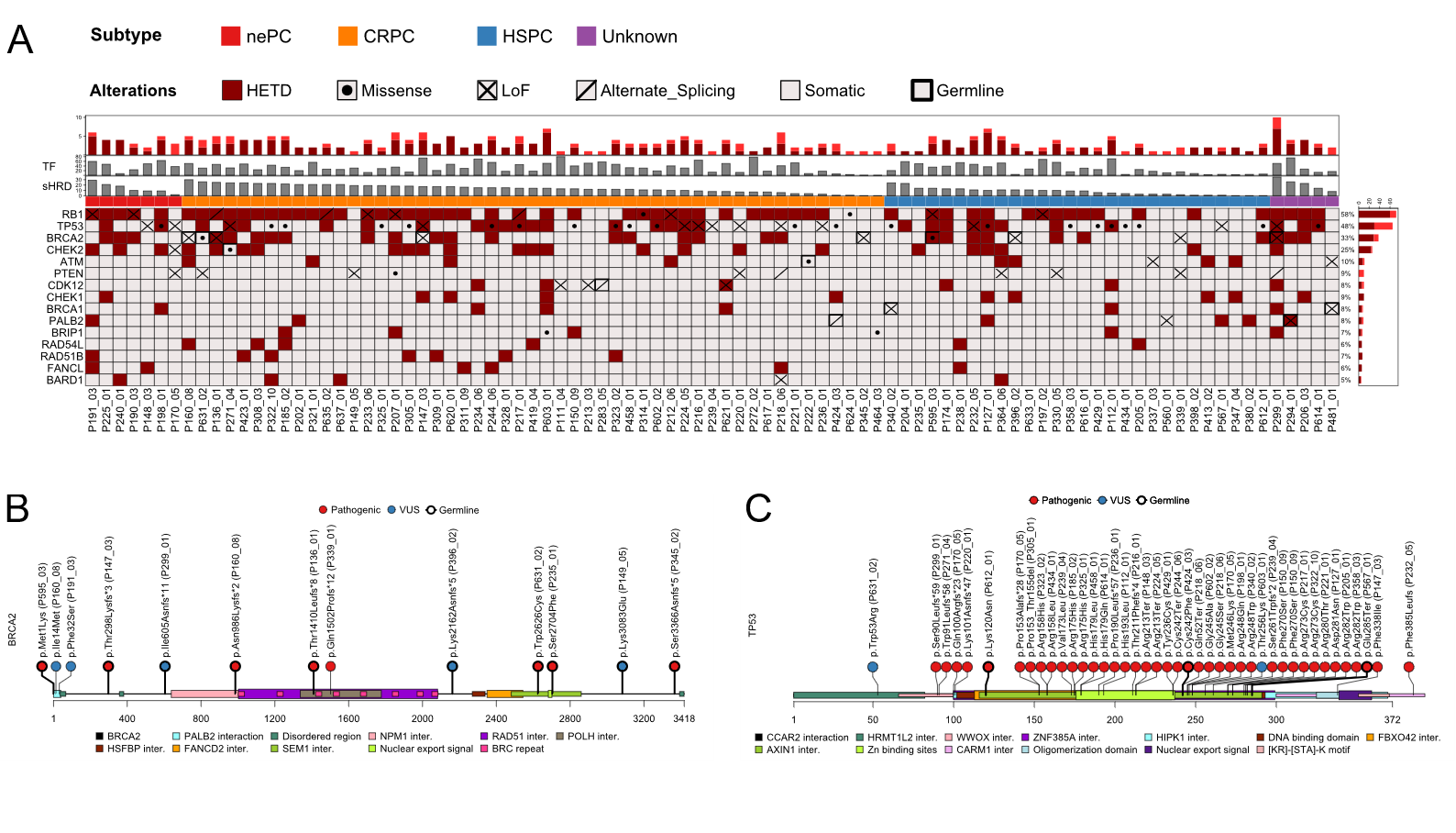
Supplementary Figure 4. Somatic landscape and mutation distribution of HRD-associated genes in plasma cfDNA. **A)** Oncoprint showing pathogenic mutations and copy number alterations across selected HRD-related genes in cfDNA from prostate cancer patients. Mutation types are annotated by shape (e.g., missense, Loss-of-function (LoF), splicing), and heterozygous deletion (HETD) is annotated by background color. Bold outline indicates a germline variant. Samples are annotated by tumor fraction (TF), sHRD score, and clinical subtype. **B)** Lollipop plot of *BRCA2* mutations annotated by protein domain. Each lollipop represents a unique somatic or germline variant, colored by pathogenicity (pathogenic: red; VUS: blue), with germline variants indicated by bold outlines. Protein interaction domains and functional regions are shown along the BRCA2 protein axis. **C)** Lollipop plot of *TP53* mutations visualized along the protein structure. Pathogenic and VUS variants are colored and labeled as in B, with structural and interaction domains annotated. Germline variants are shown with bold outlines.


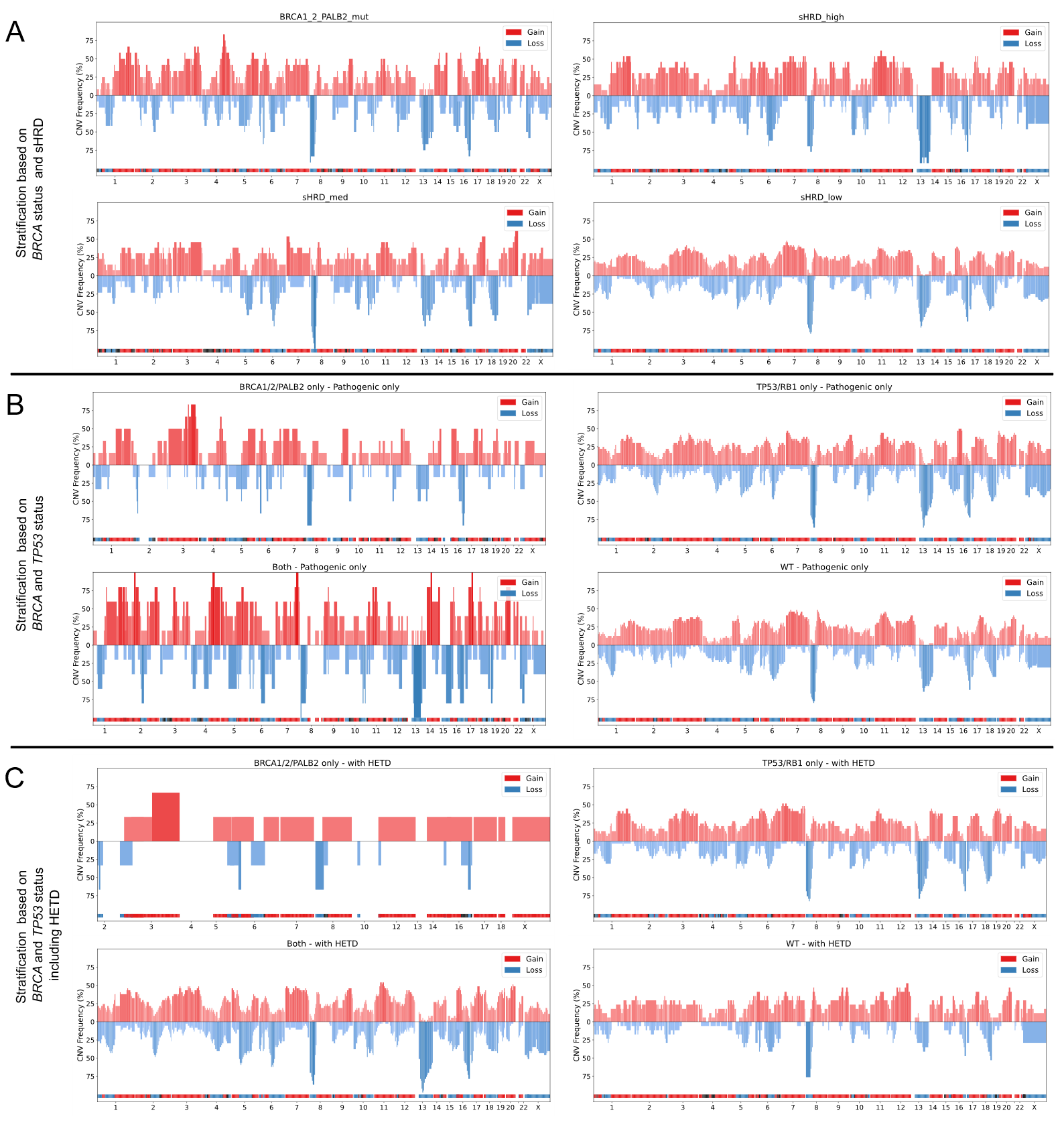
Supplementary Figure 5. Genome-wide copy number alteration frequencies across cfDNA prostate cancer subgroups. Each panel shows genome-wide copy number alteration (CNA) frequencies across 5 Mb bins along chromosomes 1–22 and X. Red bars represent the percentage of samples with copy number gains; blue bars represent copy number losses. A horizontal consensus track at the bottom of each panel marks regions with predominant gains (red), losses (blue), or neutral (gray) calls based on ≥10% frequency difference. Chromosome boundaries are indicated along the x-axis. Included samples exhibit tumor fraction (TF) > 10% A) CNA frequency plots for all high-tumor-fraction samples grouped by sHRD status (high, medium, low) and BRCA1/2/PALB2 mutation status. B) CNA profiles for subgroups defined by pathogenic mutations only, comparing BRCA1/2/PALB2-only, TP53/RB1-only, both, and wild-type (WT) cases. C) CNA profiles for the same subgroups as B, but including cases with heterozygous deletions (HETD) in the mutation group definitions.


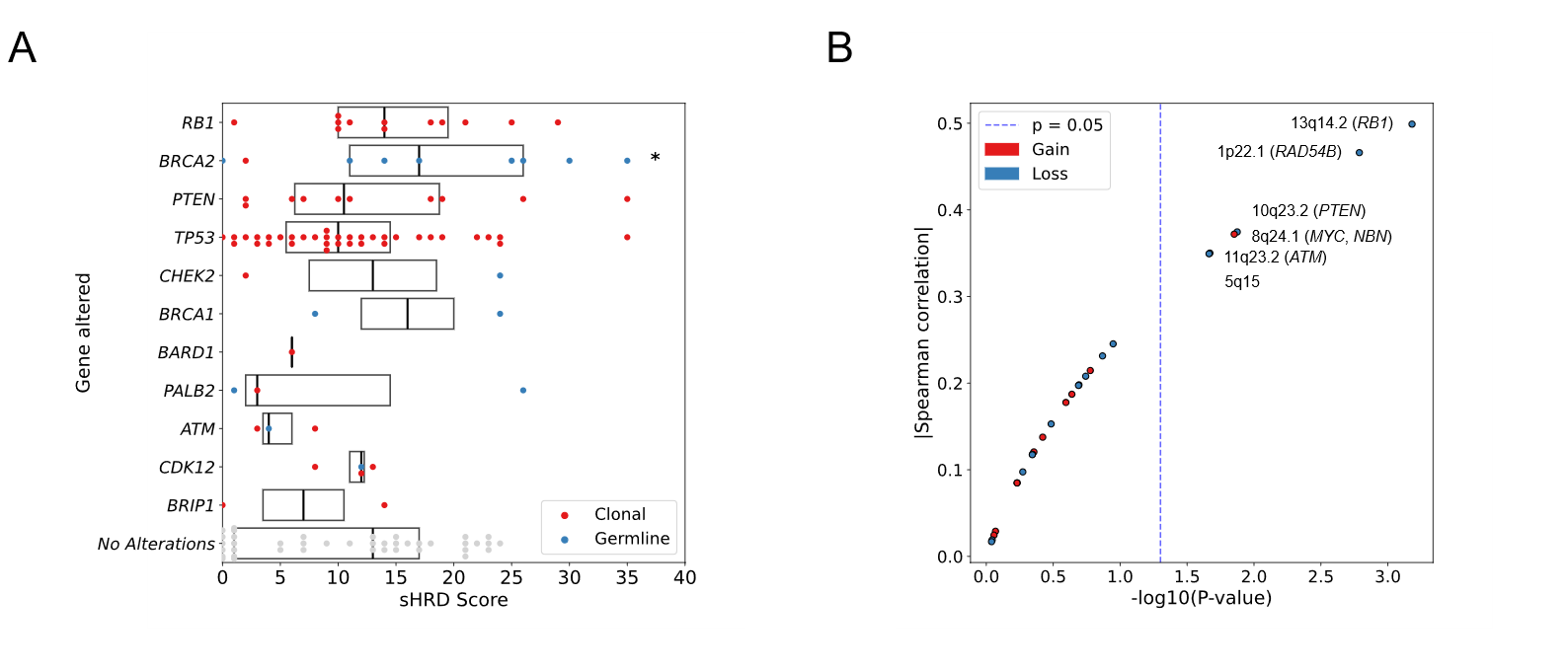
Supplementary Figure 6. Association between sHRD score and genomic alterations. **A**) Distribution of sHRD scores across samples harboring pathogenic alterations in HRR-related genes. Each point represents one sample, colored by mutation type: clonal (red) or germline (blue). Boxplots summarize the distribution of sHRD scores for each gene, and samples without any pathogenic alterations are shown as a reference group (“No Alterations”). Horizontal black line indicates the median. Asterisks indicate statistically significant differences (two-sided Mann–Whitney U test, *p* < 0.05). **B)** Volcano-style plot showing the association between focal copy number events and sHRD score across the genome. Each point represents a genomic locus, with the x-axis indicating significance (–log₁₀*p*) and the y-axis showing the absolute Spearman correlation between copy number and sHRD score. Red dots indicate gains, blue dots indicate losses. A dashed vertical line marks the p = 0.05 significance threshold.

**
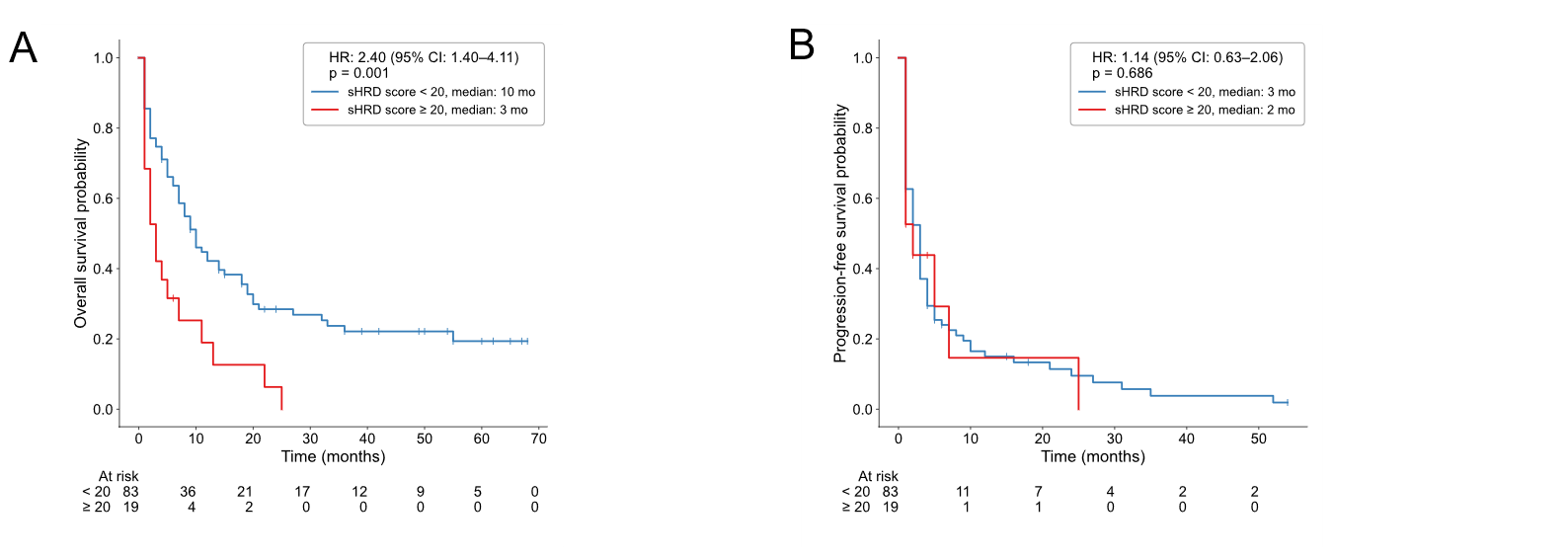
**

Supplementary Figure 7. Kaplan–Meier survival analysis stratified by genomic instability (sHRD score). **A)** Overall survival (OS) and **B)** progression-free survival (PFS) for patients with available clinical follow-up, stratified by sHRD score: low (<20, blue) vs. high (≥20, red). Median survival times are indicated in the legend for each group. Hazard ratios (HR), 95% confidence intervals (CI), and log-rank p-values were computed using Cox proportional hazards models. Censoring is marked with vertical tick marks, and at-risk counts are shown below the time axis. Survival curves were estimated using the Kaplan–Meier method.

**
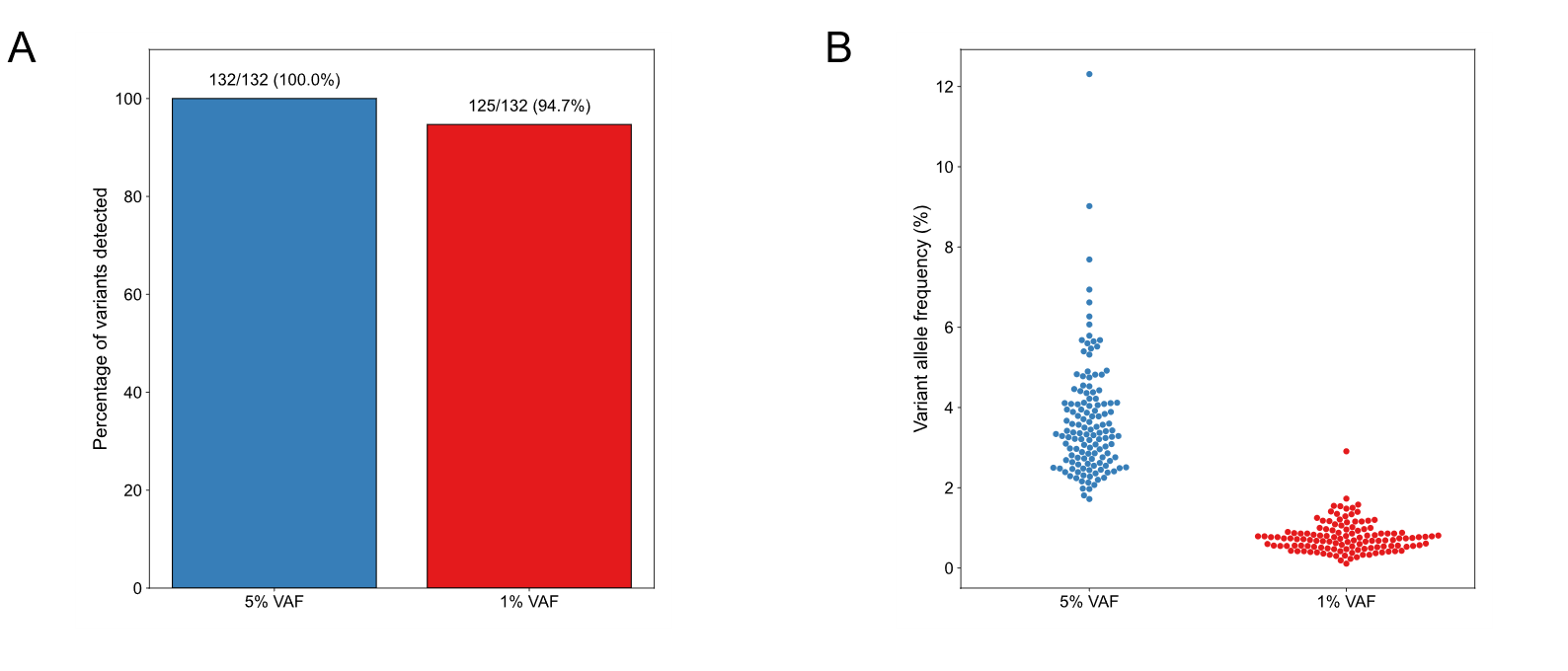
**

Supplementary Figure 8. Detection sensitivity and variant allele frequencies in a reference standard across VAF thresholds. **A)** Percentage of known variants detected in a reference material at 5% and 1% variant allele frequency (VAF), using a minimum UMI family support of 1 read. At 5% VAF, all 132/132 variants were detected (100%), while 125/132 (94.7%) were detected at 1% VAF.
**B)** Swarmplot showing the distribution of observed VAFs for all detected variants. Each point represents one variant, expressed as a percentage. Detected VAFs are consistently lower in the 1% VAF reference, as expected. Colors indicate VAF group: blue for 5% and red for 1%.

**
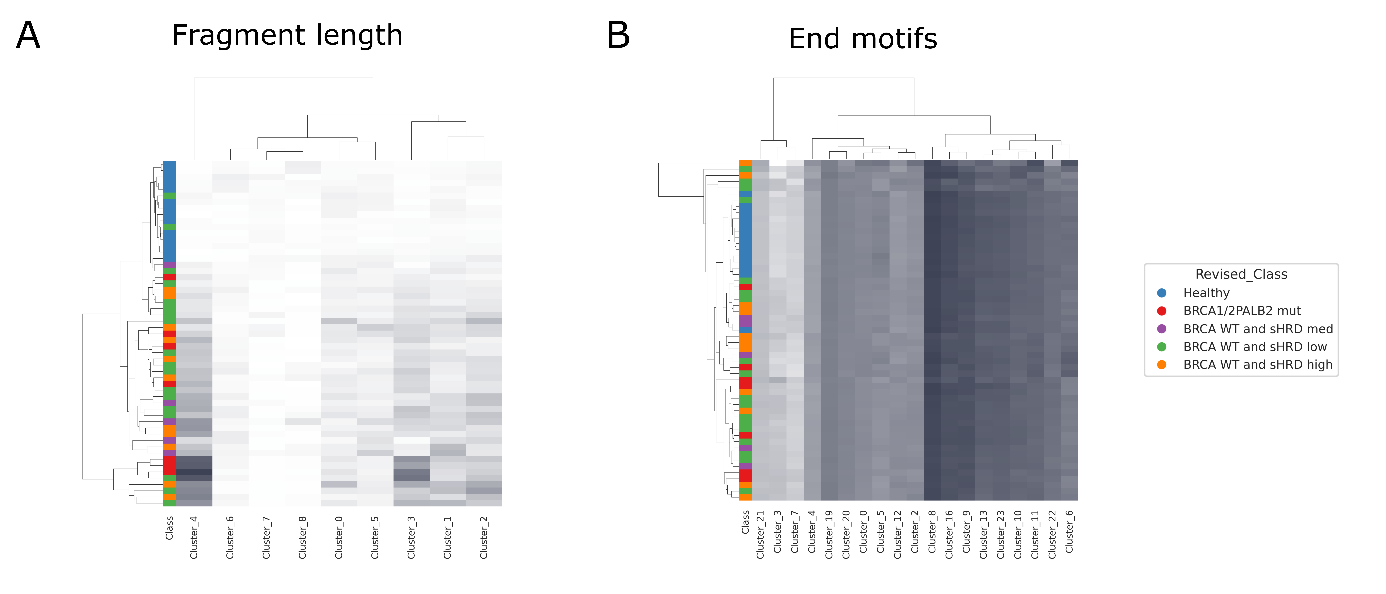
**

Supplementary Figure 9. Fragmentomic profiles stratified by HRD mutation status and genomic instability. **A)** Heatmap of cfDNA fragment length distributions across patient and control samples. Rows represent individual plasma samples, and columns correspond to fragment length bins. Values are normalized intensities. **B)** Heatmap of cfDNA 4-mer end motif usage from the same samples. Columns represent specific end motifs, and color intensity reflects relative motif frequency.
Samples are hierarchically clustered in both panels. Sample classes are annotated by color: red (pathogenic mutation in *BRCA1/2* or *PALB2*), yellow (no mutation, sHRD ≥ 20), orange (no mutation, intermediate sHRD 15–20), gray (no mutation, sHRD < 15), and green (healthy controls).


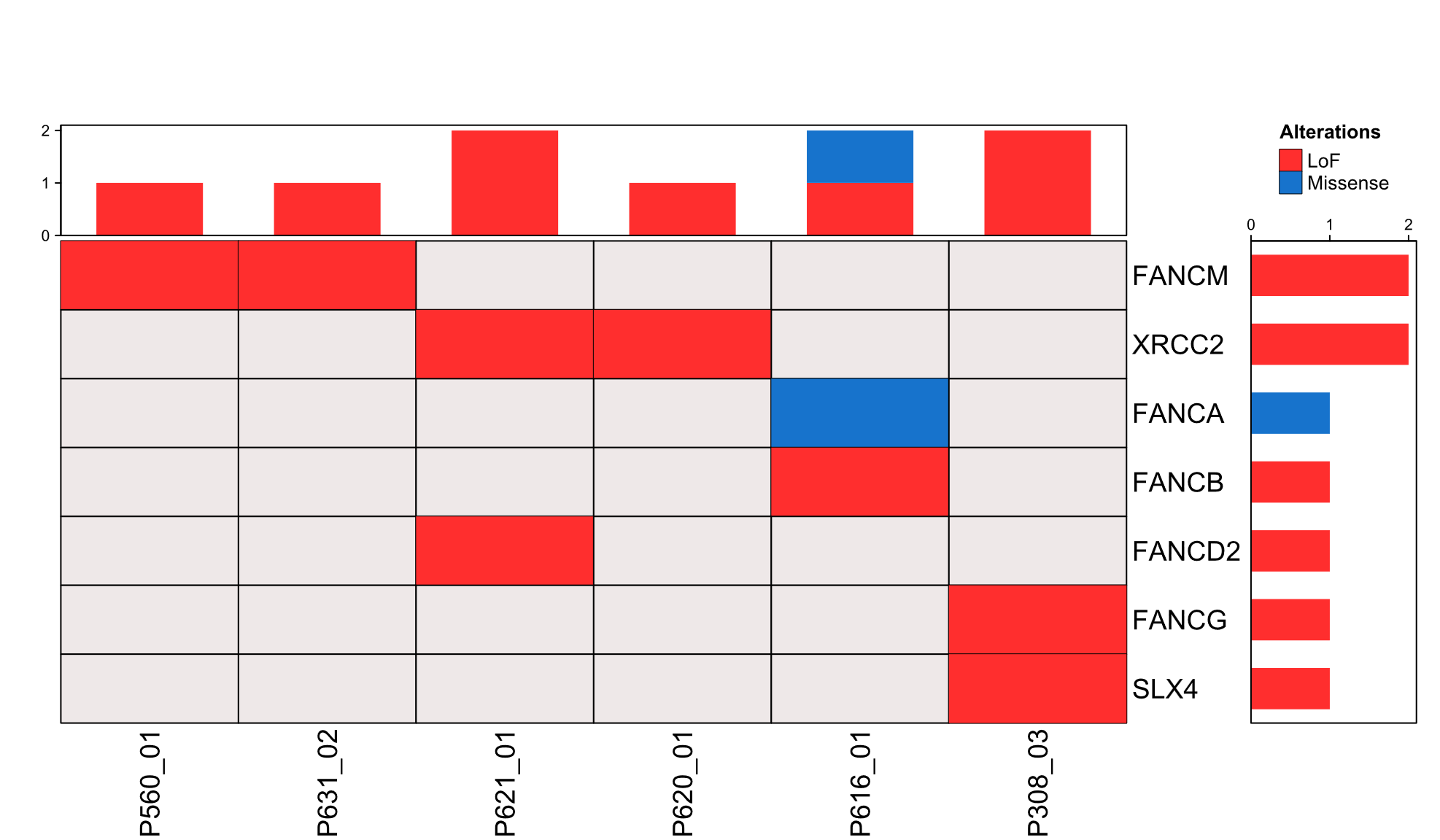


Supplementary Figure 10. Somatic landscape of Fanconi anemia (FANC) pathway genes in prostate cancer WES data. Oncoprint summarizing pathogenic and likely pathogenic mutations identified in Fanconi pathway genes across the WES dataset. Each column represents a patient, and each row corresponds to a gene. The top barplot indicates the number of mutated genes per patient, and the right barplot shows the number of patients affected per gene.


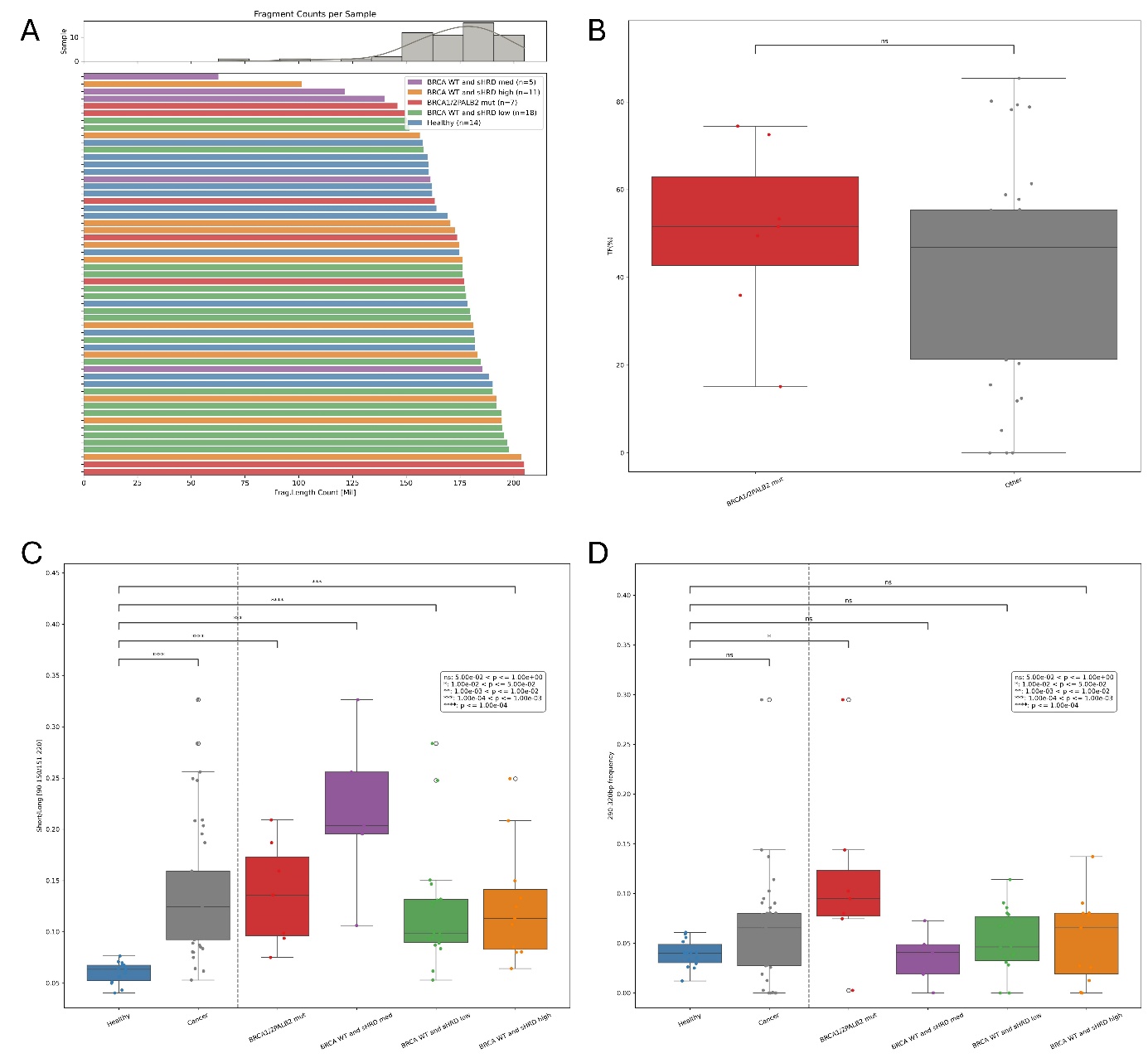
Supplementary Figure 11. Fragment length distributions stratified by HRR mutation status. **(A)** Fragment counts across all samples, demonstrating balanced sequencing depth between groups. **(B)** Tumor fraction comparison between HRR-mutated (*BRCA1/2* or *PALB2*) and HRR-wildtype tumors, showing no significant difference. **(C)** Enrichment of short fragments in all cancer groups relative to healthy controls. **(D)** Specific enrichment of 290–320 bp fragments observed exclusively in BRCA1/2 or PALB2 mutation samples, consistent with dinucleosome-associated lengths. Adjusted p-values were obtained from two-sided Mann–Whitney U tests with Benjamini–Hochberg false-discovery-rate correction, as described in the Methods.


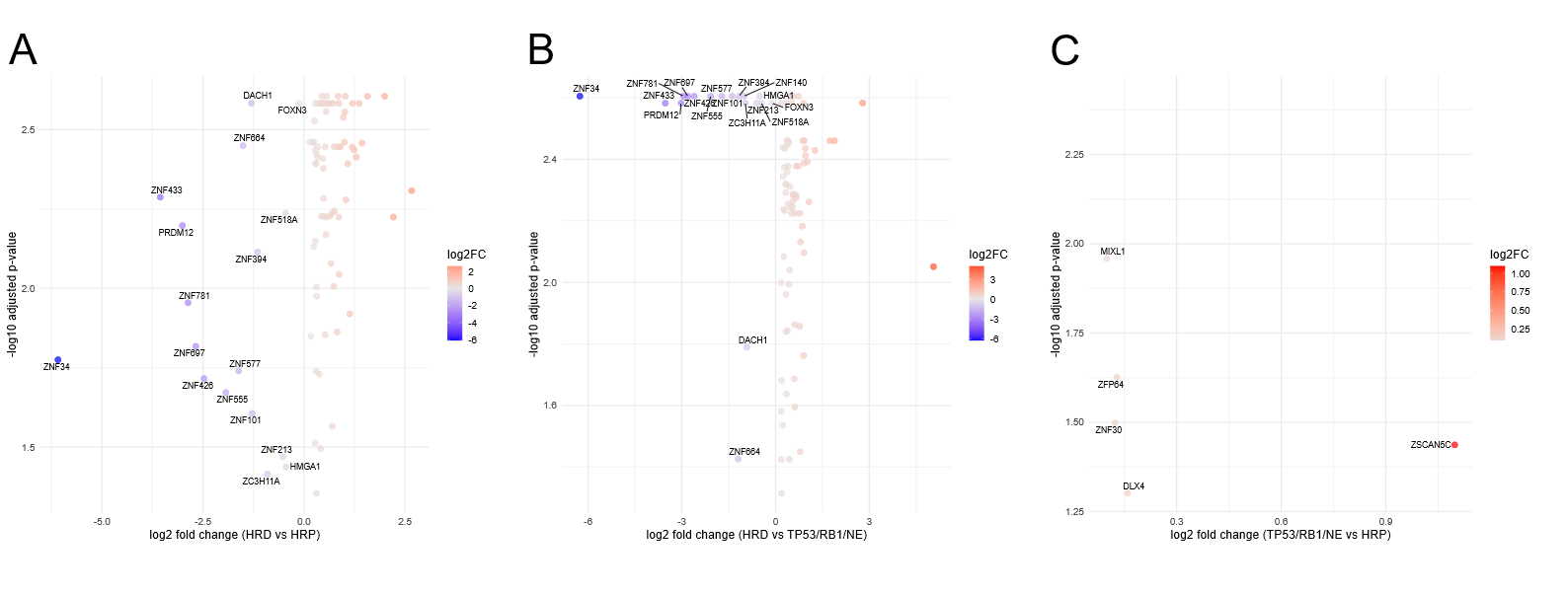
Supplementary Figure 12. Quantification of differential accessibility transcription factor binding sites. **(A)** *BRCA2*/sHRD-high versus HRR-proficient tumors, **(B)** *BRCA2*/sHRD-high versus *TP53*/*RB1*/NE tumors, and **(C)** *TP53*/*RB1*/NE versus HRR-proficient tumors. Each panel shows log₂ fold change (x-axis) versus –log₁₀ adjusted p-value (y-axis) for chromatin accessibility at ZNF TFBS derived from cfDNA. Adjusted p-values were obtained from two-sided Mann–Whitney U tests with Benjamini–Hochberg false-discovery-rate correction, as described in the Methods. Each point represents a single transcription factor binding site, with color indicating the direction and magnitude of the log₂ fold change. Gene names are labeled for significantly altered loci. Together, these comparisons highlight consistent reductions in accessibility at specific ZNF TFBS in HRD cfDNA, with the most pronounced effects observed in *BRCA2*/sHRD-high cases relative to *TP53*/*RB1*/*NE* tumors.


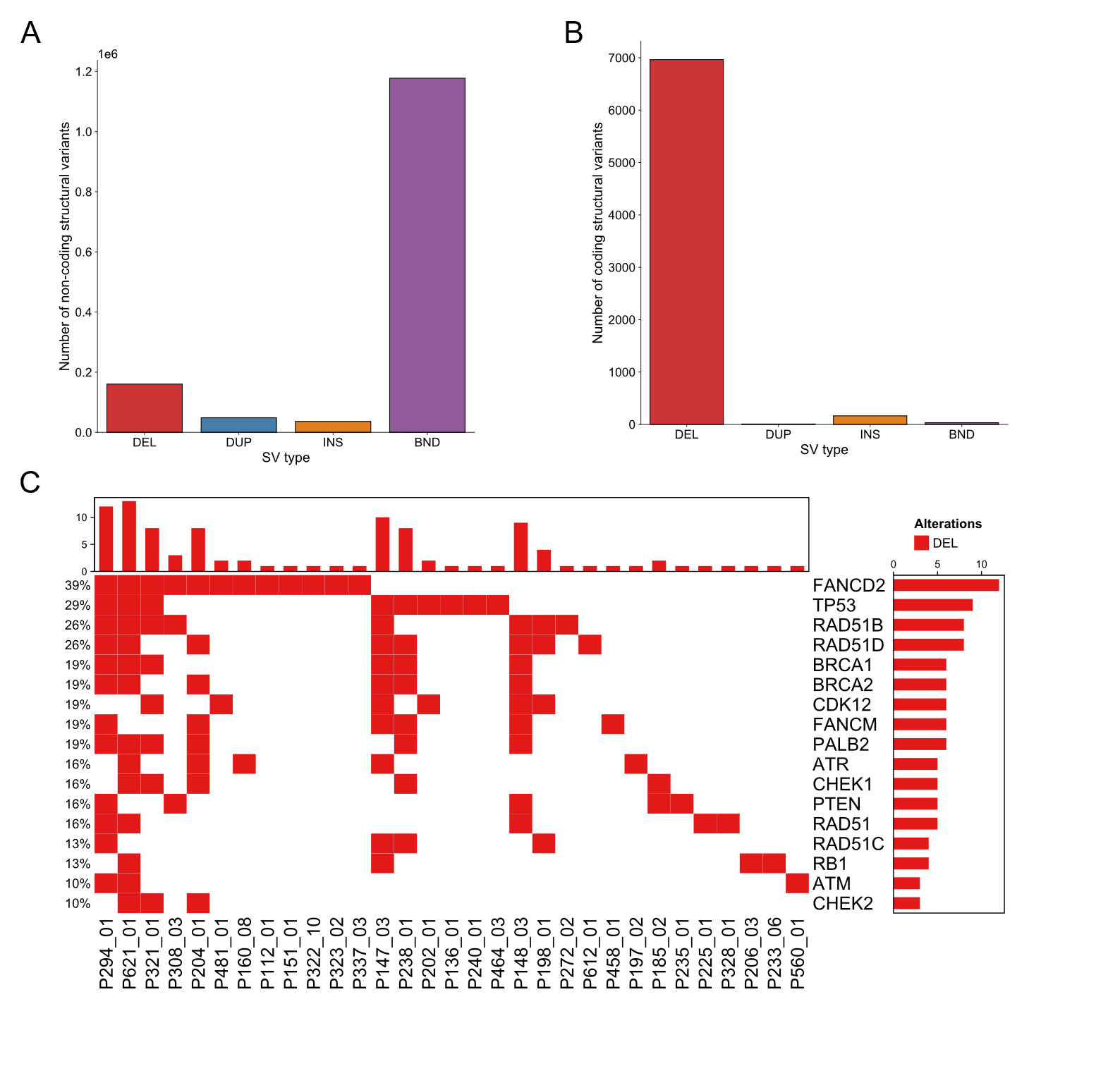
Supplementary Figure 13. Structural variant landscape in cfDNA WGS samples. **(A)** Total number of structural variants per type (deletions, duplications, insertions, breakends) detected across in the high-coverage WGS samples. **(B)** Subset of structural variants affecting coding regions only, showing the predominance of large deletions. **(C)** Oncoprint of homologous recombination repair (HRR) genes affected by structural variants. Each cell represents the presence of a deletion (red), duplication (blue), insertion (orange), or translocation/breakend (purple). Deletions in *BRCA1/2*, *PALB2*, *RAD51* family members, and other HRR regulators were common, while no recurrent fusions were detected. Duplications, insertions, or other structural variants were not observed in HRR genes.

References

1. Heitzer E, Auer M, Hoffmann EM, Pichler M, Gasch C, Ulz P, et al. Establishment of tumor‐specific copy number alterations from plasma DNA of patients with cancer. Intl Journal of Cancer. 2013 July 15;133(2):346–56.

2. Belic J, Koch M, Ulz P, Auer M, Gerhalter T, Mohan S, et al. Rapid Identification of Plasma DNA Samples with Increased ctDNA Levels by a Modified FAST-SeqS Approach. Clinical Chemistry. 2015 June 1;61(6):838–49.

3. Md V, Misra S, Li H, Aluru S. Efficient Architecture-Aware Acceleration of BWA-MEM for Multicore Systems [Internet]. arXiv; 2019 [cited 2025 Oct 24]. Available from: https://arxiv.org/abs/1907.12931

4. Xu C, Gu X, Padmanabhan R, Wu Z, Peng Q, DiCarlo J, et al. smCounter2: an accurate low-frequency variant caller for targeted sequencing data with unique molecular identifiers. Bioinformatics. 2019 Apr 15;35(8):1299–309.

5. Liu X, Jian X, Boerwinkle E. dbNSFP v2.0: A Database of Human Non-synonymous SNVs and Their Functional Predictions and Annotations. Human Mutation. 2013 Sept 1;34(9):E2393–402.

6. Karczewski KJ, Francioli LC, Tiao G, Cummings BB, Alföldi J, Wang Q, et al. The mutational constraint spectrum quantified from variation in 141,456 humans. Nature. 2020;581(7809):434–43.

7. Richards S, Aziz N, Bale S, Bick D, Das S, Gastier-Foster J, et al. Standards and guidelines for the interpretation of sequence variants: a joint consensus recommendation of the American College of Medical Genetics and Genomics and the Association for Molecular Pathology. Genetics in Medicine. 2015 May 1;17(5):405–24.

8. Sheldon M. R. Introduction to Probability Models. Elsevier; 2014.

9. Blokzijl F, Janssen R, van Boxtel R, Cuppen E. MutationalPatterns: comprehensive genome-wide analysis of mutational processes. Genome Med. 2018 Apr 25;10(1):33.

10. Alexandrov LB, Kim J, Haradhvala NJ, Huang MN, Tian Ng AW, Wu Y, et al. The repertoire of mutational signatures in human cancer. Nature. 2020 Feb 1;578(7793):94–101.

11. Favero F, Joshi T, Marquard AM, Birkbak NJ, Krzystanek M, Li Q, et al. Sequenza: allele-specific copy number and mutation profiles from tumor sequencing data. Annals of Oncology. 2015 Jan;26(1):64–70.

12. Heitzer E, Ulz P, Belic J, Gutschi S, Quehenberger F, Fischereder K, et al. Tumor-associated copy number changes in the circulation of patients with prostate cancer identified through whole-genome sequencing. Genome Medicine. 2013;5(4):30.

13. Broad Institute. Picard Toolkit [Internet]. Broad Institute, GitHub repository; 2018. Available from: http://broadinstitute.github.io/picard/

14. Adalsteinsson VA, Ha G, Freeman SS, Choudhury AD, Stover DG, Parsons HA, et al. Scalable whole-exome sequencing of cell-free DNA reveals high concordance with metastatic tumors. Nature communications. 2017;8(1):1324.

15. Kent WJ, Sugnet CW, Furey TS, Roskin KM, Pringle TH, Zahler AM, et al. The human genome browser at UCSC. Genome Res. 2002 June;12(6):996–1006.

16. Eeckhoutte A, Houy A, Manié E, Reverdy M, Bieche I, Marangoni E, et al. ShallowHRD: detection of homologous recombination deficiency from shallow whole genome sequencing. Bioinformatics. 2020;36(12):3888–9.

17. Chen X, Schulz-Trieglaff O, Shaw R, Barnes B, Schlesinger F, Källberg M, et al. Manta: rapid detection of structural variants and indels for germline and cancer sequencing applications. Bioinformatics. 2016 Apr 15;32(8):1220–2.

18. McLaren W, Gil L, Hunt SE, Riat HS, Ritchie GRS, Thormann A, et al. The Ensembl Variant Effect Predictor. Genome Biol. 2016 Dec;17(1):122.

19. Lazzeri I, Spiegl BG, Hasenleithner SO, Speicher MR, Kircher M. LBFextract: Unveiling transcription factor dynamics from liquid biopsy data. Computational and Structural Biotechnology Journal. 2024 Dec 1;23:3163–74.

20. Spiegl B, Kapidzic F, Röner S, Kircher M, Speicher MR. GCparagon: evaluating and correcting GC biases in cell-free DNA at the fragment level. NAR Genomics and Bioinformatics. 2023 Oct 11;5(4):lqad102.
